# A comprehensive review/expert statement on environmental risk factors of cardiovascular disease

**DOI:** 10.1093/cvr/cvaf119

**Published:** 2025-08-11

**Authors:** Thomas Münzel, Mette Sørensen, Jos Lelieveld, Philip J Landrigan, Marin Kuntic, Mark Nieuwenhuijsen, Mark R Miller, Alexandra Schneider, Andreas Daiber

**Affiliations:** Department of Cardiology, University Medical Center Mainz, Johannes Gutenberg University, Langenbeckstrasse 1, Mainz 55131, Germany; Work, Environment and Cancer, Danish Cancer Institute, Copenhagen, Denmark; Department of Natural Science and Environment, Roskilde University, Roskilde, Denmark; Atmospheric Chemistry Department, Max Planck Institute for Chemistry, Mainz, Germany; Global Observatory on Planetary Health, Boston College, Boston, USA; Centre Scientifique de Monaco, City of Monaco, MC, Monaco; Department of Cardiology, University Medical Center Mainz, Johannes Gutenberg University, Langenbeckstrasse 1, Mainz 55131, Germany; Institute for Global Health (ISGlobal), Barcelona, Spain; Department of Experimental and Health Sciences, Universitat Pompeu Fabra (UPF), Barcelona, Spain; CIBER Epidemiología y Salud Pública (CIBERESP), Madrid, Spain; Centre for Cardiovascular Science, University of Edinburgh, Edinburgh, United Kingdom; Institute of Epidemiology, Helmholtz Zentrum München—German Research Center for Environmental Health (GmbH), Neuherberg, Germany; Department of Cardiology, University Medical Center Mainz, Johannes Gutenberg University, Langenbeckstrasse 1, Mainz 55131, Germany; German Centre for Cardiovascular Research (DZHK), Partner Site Rhine-Main, Mainz, Germany

**Keywords:** Environment, Air pollution, Noise exposure, Soil and water pollution, Chemical pollution, Oxidative stress, Endothelial dysfunction

## Abstract

Cardiovascular disease (CVD) is the leading cause of mortality globally, with over 20 million deaths each year. While traditional risk factors—such as hypertension, diabetes, smoking, and poor diet—are well-established, emerging evidence underscores the profound impact of environmental exposures on cardiovascular health. Air pollution, particularly fine particulate matter (PM_2.5_), contributes to approximately 8.3 million deaths annually, with over half attributed to CVD. Similarly, noise pollution, heat extremes, toxic chemicals, and light pollution significantly increase the risk of CVD through mechanisms involving oxidative stress, inflammation, and circadian disruption. Recent translational and epidemiological studies show that chronic exposure to transport noise increases the risk of myocardial infarction, stroke, and heart failure. Air pollution, even below regulatory thresholds, promotes atherosclerosis, vascular dysfunction, and cardiac events. Novel threats such as micro- and nano-plastics are emerging as contributors to vascular injury and systemic inflammation. Climate change exacerbates these risks, with heatwaves and wildfires further compounding the cardiovascular burden, especially among vulnerable populations. The cumulative effects of these exposures—often interacting with behavioural and socioeconomic risk factors—are inadequately addressed in current prevention strategies. The exposome framework offers a comprehensive approach to integrating lifelong environmental exposures into cardiovascular risk assessment and prevention. Mitigation requires systemic interventions including stricter pollution standards, noise regulations, sustainable urban design, and green infrastructure. Addressing environmental determinants of CVD is essential for reducing the global disease burden. This review calls for urgent policy action and for integrating environmental health into clinical practice to safeguard cardiovascular health in the Anthropocene.

## Graphical Abstract

**Graphical Abstract:**
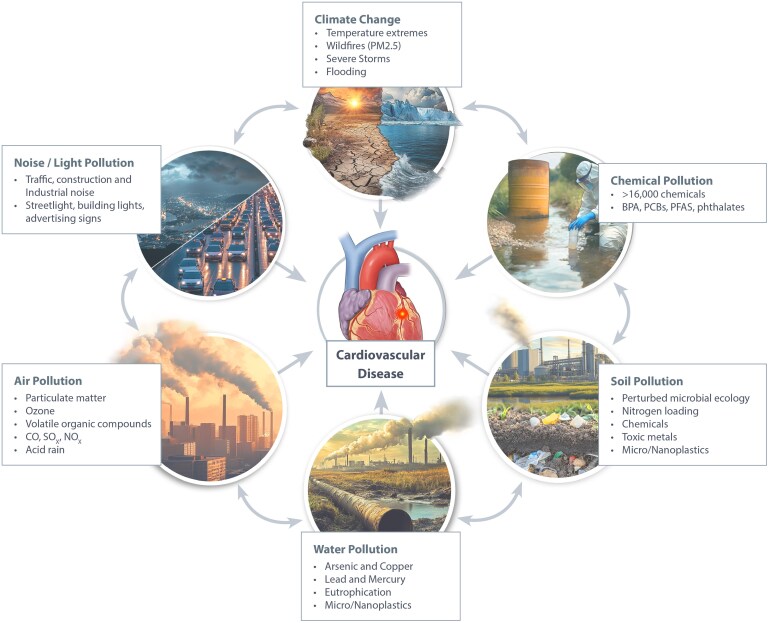

## 1. Introduction: GBD and the environment

Cardiovascular diseases (CVD), including coronary artery disease (CAD), heart failure, arrhythmias, stroke, and arterial hypertension, affected over half a billion people worldwide in 2021 and were responsible for 20.5 million deaths—nearly one-third of all global fatalities.^1^ Modifiable risk factors that contributed to excess CVD deaths globally were low physical activity (0.4 million deaths), high body mass index (2.0 million deaths), high fasting plasma glucose (2.3 million deaths), elevated LDL cholesterol (3.8 million deaths), tobacco use (8.0 million deaths), and elevated blood pressure (10.8 million deaths).

Cardiovascular disease (CVD)—the leading cause of death in the EU and globally—affects more than 60 million Europeans, accounts for over 1.7 million deaths annually on the continent, and costs its economy an estimated €282 billion each year.^2^ The burden is not evenly distributed across the continent, as morbidity and mortality rates are generally higher in central and eastern Europe compared with northern and western regions. Over the past decades, improved screening, treatment, and lifestyle changes, such as reduced smoking, have led to significant declines in CVD mortality across European countries. However, CVD incidence has not declined as much, mainly due to an ageing population and the persistent influence of modifiable risk factors.

**Figure 1:**
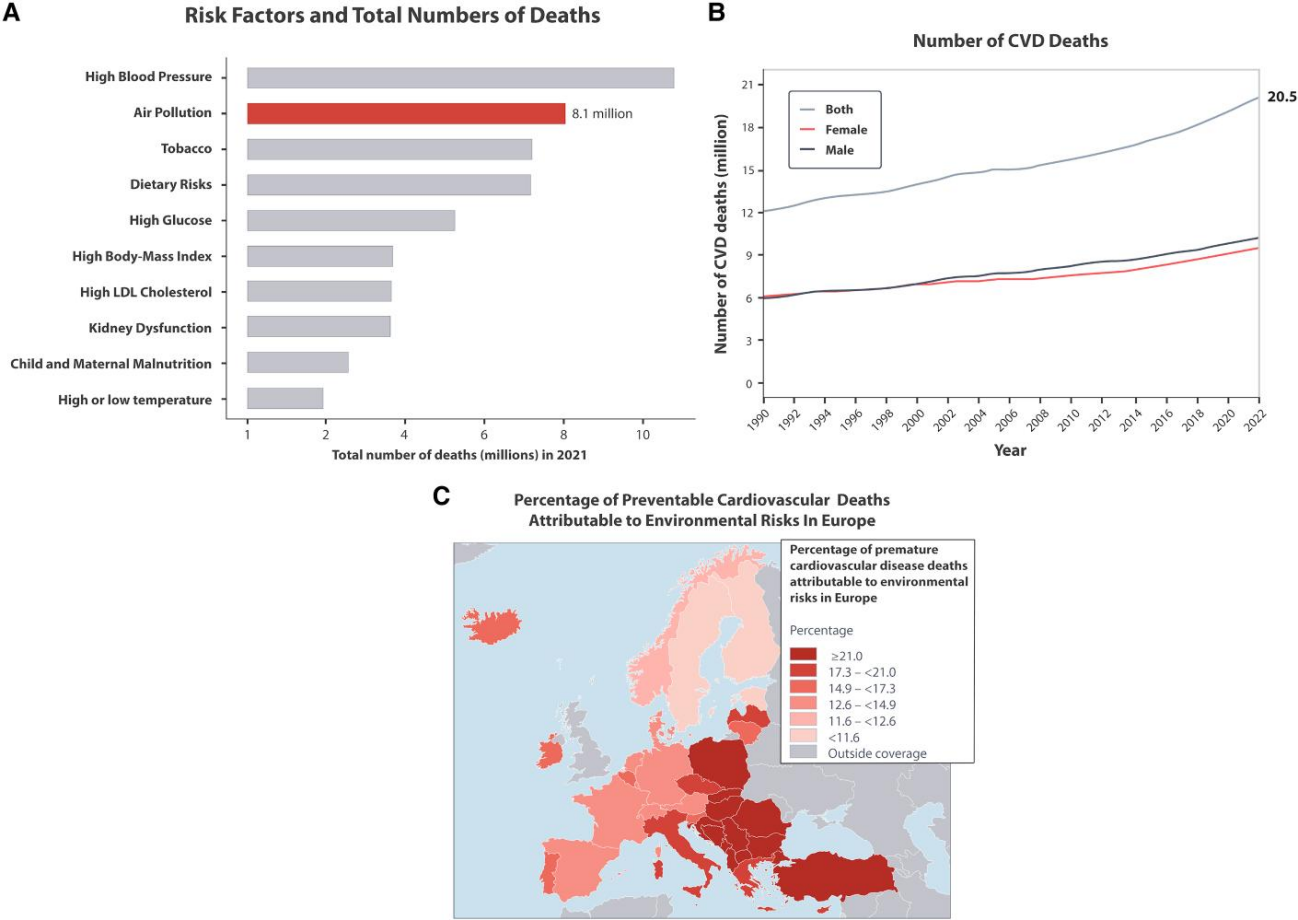
The burden of CVD deaths and key risk factors. (*A*): Significant cardiovascular risk factors, with high blood pressure as the leading cause, followed by air pollution, responsible for 8.1 million deaths. LDL, low-density lipoprotein. (*B*): the steady increase in CVD deaths, reaching 20.5 million in 2021. (*C*): The environmental contribution to CVD in Europe is striking, with an estimated 18% of all CVD-related deaths attributed to these factors. Panel (*A*) is based on data of the GBD Study and adapted from a report of the Health Effects Institute in 2024 with permission.^6^ Panel (*B*) is adapted from the World Heart Report 2023.^7^ Panel C reproduced with permission from.^5^

Traditionally, efforts to prevent CVD have focused on well-established clinical and behavioural risk factors, including high blood pressure, high LDL cholesterol, excess weight, diabetes, tobacco use, physical inactivity, and unhealthy diets. While these risk factors remain critically important, growing evidence highlights the substantial contribution of environmental factors to CVD. Air pollution, extremes of heat and cold, noise, and toxic chemicals—especially lead—are increasingly recognized as key environmental contributors.^3,4^ These factors do not operate in isolation but interact with clinical and behavioural risk factors and socioeconomic determinants such as low income, education, and job insecurity, further compounding their effects on vulnerable populations. The environmental contribution to CVD in Europe is striking, with an estimated 18% of all CVD-related deaths attributed to these factors^5^ (*Figure 1*). This figure is likely a strong underestimate of the environmental contribution to CVD, as current calculations often omit workplace exposures, the effects of environmental noise, and other toxic chemicals beyond lead.^8^ Air pollution remains the most significant environmental risk, especially fine particulate matter [particulate matter (PM) with a diameter of 2.5 micrometres or less; PM_2.5_]. In 2021, air pollution was estimated to contribute to 8.3 million deaths globally, making up about 12% of total deaths.^9^ PM_2.5_ alone accounted for 7.9 million deaths,^9^ more than 90% of the total air pollution disease burden.

In addition, a recent analysis established that 5.545 million [95% Confidence Interval (CI): 2.305–8.271 million] adults died from CVD in 2019 just due to lead exposure.^10^

Importantly, concerning the risk factors for death, ambient air pollution is now ranked number 2 (*Figure 1*), just surpassed by the leading risk factor, arterial hypertension.^6^ However, in terms of disability-adjusted life years (DALYs), air pollution has been the number one contributing factor to the global disease burden substantially for decades.^11^

Yet, despite these staggering statistics, pollution reduction has received limited attention in cardiovascular prevention programmes. Most policies continue to focus primarily on individual lifestyle choices, mainly neglecting the broader environmental context. Given the scale of pollution-related CVD deaths, addressing environmental risks must become a central pillar of cardiovascular prevention strategies.

Unlike behavioural risk factors, which individuals can modify to some extent, ecological exposures often require systemic, population-wide interventions to be effective. Government policies to reduce pollution, mitigate climate change, and enforce stricter environmental regulations could profoundly benefit public health. Adapting to climate change, improving air quality, and minimising exposure to hazardous chemicals and noise pollution would reduce CVD incidence and yield co-benefits for overall health and well-being. A comprehensive approach integrating environmental risk reduction into traditional prevention strategies is essential for effectively combating CVD in the coming decades.

This in-depth expert review will examine the epidemiology and pathophysiology of environmental stressors (although we exclude the impact of environmental health risk factors such as mental stress^12^ and ionising radiation, either from anticancer therapy^13^ or ionospheric and geomagnetic exposures^14^ as beyond the present scope). We will explore potential solutions and mitigation strategies to reduce environmental stressors’ adverse health effects, with a particular emphasis on CVD.

## 2. Air pollution

### 2.1 Sources in the anthropocene

Air pollutants have been recognized since ancient times, but their sources and composition have significantly changed with industrialisation and urbanisation, most markedly in the Anthropocene, the current geologic epoch in which humankind has become the dominant influence on planetary systems. Modern anthropogenic pollutants, many of which are derived from combustion processes, are now a critical public health concern.^15,16^ The characteristics of air pollution result from complex chemical reactions in the atmosphere in addition to emissions from various sources. This complexity necessitates new classification criteria for fine particles, focusing not only on size or mass, but also on properties such as surface reactivity and contamination with hazardous substances, polycyclic aromatic hydrocarbons (PAHs) or microbial pathogens. Furthermore, there is a need to reflect the chemical properties of airborne particles as they change with age and during atmospheric transport.^17,18^

Urban pollutants consist of gaseous compounds like ozone (O_3_), nitrogen oxides (NOx = NO + NO_2_), volatile organic compounds (VOCs, e.g. benzene, toluene, aldehydes), carbon monoxide (CO), and sulfur dioxide (SO_2_). In the atmosphere, these gases react photochemically, and the lower-volatility products can form PM_2.5_, which is typically a mixture of organic and inorganic compounds.^19^ There is extensive mechanistic and epidemiological evidence that PM_2.5_ is a major contributor to morbidity and mortality.^20,21^ The particles can partially be directly emitted as PM_2.5_, such as black and organic carbon, in addition to larger ones, including mineral dust, PM_10_ (PM with a diameter of <10 µm), or ultrafine particles (UFPs; or PM_0.1_, with a diameter <0.1 μm). Nanosized UFPs may be particularly harmful to cardiovascular health due to their small size, reactive chemical composition (e.g. pro-oxidative properties), large surface-to-mass ratio, and ability for these particles to penetrate alveoli, enter the circulation, and directly damage multiple organs, thus having implications for many different diseases.^22,23^

While CO is toxic at very high concentrations, such levels are uncommon in ambient air. However, due to its ability to displace oxygen in haemoglobin of blood cells, leading to oxygen deprivation in organs, chronic exposure of lower concentrations has been linked to adverse health effects, including CVD.^24^ Pollutants like NO_2_, O_3_, and PM cause oxidative stress in tandem with inflammatory responses.^25,26^

These oxidative pollutants generate reactive oxygen species (ROS), often catalytically, emphasising the need for comprehensive toxicological, modelling, and epidemiological studies.^27–30^ Ozone (O_3_), a highly reactive molecule with strong oxidising power, contributes to systemic oxidative stress by generating ROS such as hydrogen peroxide (H_2_O_2_),and an irritant to the respiratory system, exacerbating asthma, while long-term exposure causes chronic obstructive pulmonary disease (COPD).^31^ NO_2_ is also formed in the atmosphere, via the reaction between NO and oxygen, O_3_ or VOCs with NO, which is directly emitted by fossil fuel combustion in traffic and energy generation. NO_2_ irritates the airways and causes asthma and other respiratory diseases, but has also been associated with CVDs, various other health conditions and mortality.^32^

Climate change significantly influences air pollution, as hot weather and intense solar radiation in cloud-free conditions increase the formation of reactive air pollutants. Conversely particularly black carbon, ozone, and methane (CH_4_; a relatively long-lived VOC) contribute to global warming, creating a feedback loop that exacerbates cardiovascular health risks. Therefore, simultaneously mitigation of air pollution and climate change, which have many common emission sources (notably from fossil fuel combustion), is a leading option to improve public health and will produce a co-benefit, or double benefit.^33–35^ A recent evaluation of 1500 climate policies worldwide in the past decades identified only 63 effective interventions (<5%) that have led to significant emission reductions of greenhouse gases. This review identifies stringent air pollution standards as one of the most successful interventions.^36^

### 2.2 Global burden of disease

Air pollution poses a significant health risk, contributing to disease and excess deaths on a global scale. According to the World Health Organization (WHO), both gaseous and particulate pollutants are significant factors for respiratory infections, COPD, lung cancer, and cardiovascular conditions such as heart attacks and strokes.^37^

Chronic exposure to air pollution is of particular concern due to its link to non-communicable diseases (NCDs), which are being investigated through epidemiological cohort studies performed in many countries. Health risks have been identified even at PM concentrations well below the annual PM_2.5_ and PM_10_ limits recommended by European guidelines. In 2021, the WHO decreased the guideline annual concentration of PM_2.5_ from 10 to 5 µg/m^2^, a level below which adverse health impacts may be expected, though they are considered not yet proven.^38^ While the European Union has proposed a new PM_2.5_ limit of 10 µg/m^3^ annually, the legal limit remains 25 µg/m^3^.

Exposure of the global population to PM_2.5_ and O_3_ can be estimated with satellite and ground-based measurements and data-informed modelling.^39^ The FUSION risk model was developed to assess health outcomes and utilizes hazard ratio functions based on many cohort studies performed in various countries.^40^ Results include excess mortality rates and years of life lost from six disease categories: lower respiratory tract infections, COPD, ischaemic heart disease (IHD), cerebrovascular diseases (e.g. strokes), diabetes type 2, lung cancer, and another category that covers non-accounted NCDs (this category is the residual between all-cause and disease-specific mortality attributable to air pollution, including neurological disorders and hypertension^41^).^9^

A recent Global Burden of Disease (GBD) study identified PM as the leading specific health risk factor, contributing 8% to the total loss of DALYs (the sum of years of life lost and the years lived in disability), followed by high systolic blood pressure, smoking, low birthweight and short gestation, and high fasting plasma glucose.^11^

**Figure 2:**
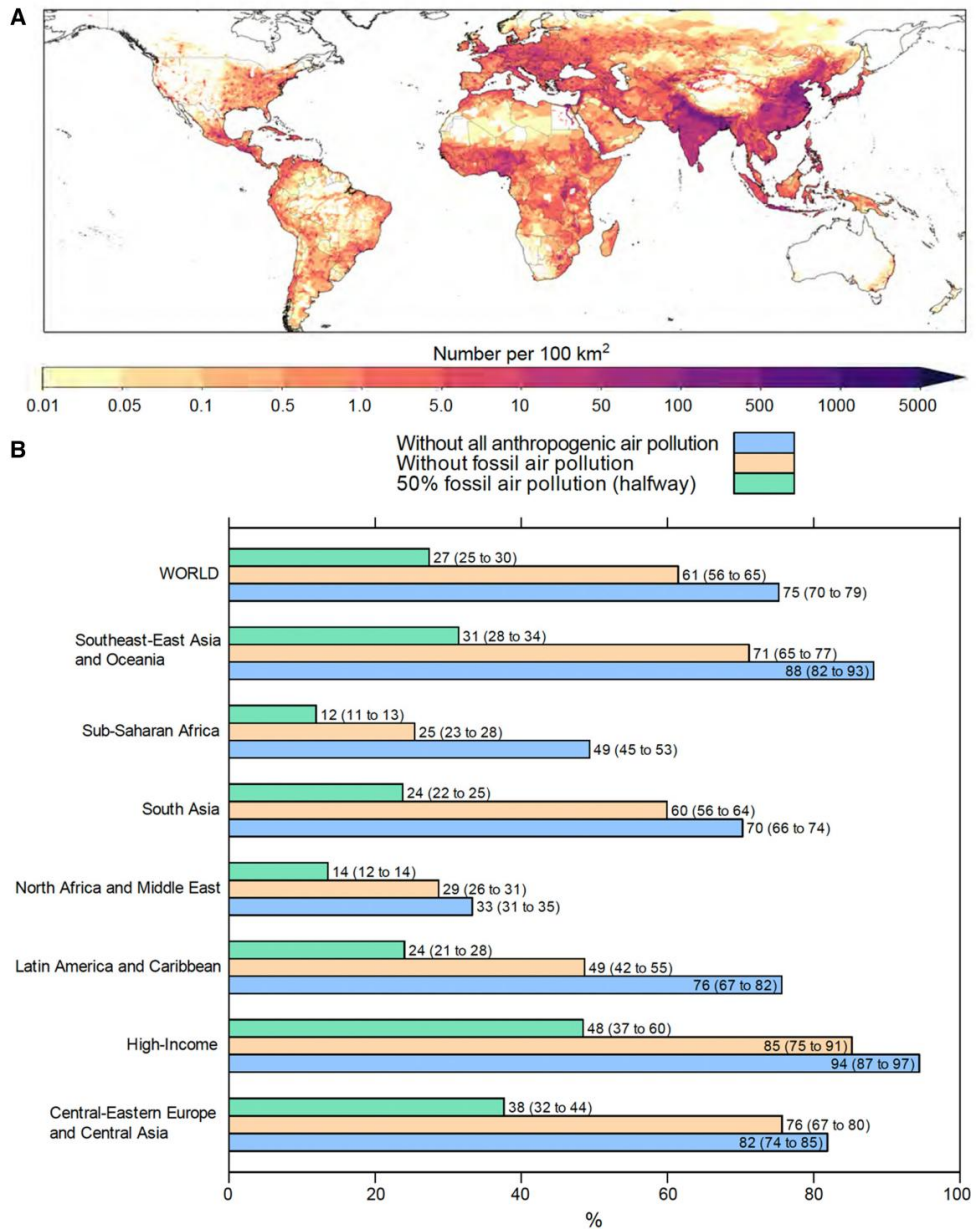
Annual all-cause excess deaths attributable to ambient fine particulate matter (PM_2.5_) and ozone (O_3_). (*A*) Excess mortality attributable to long-term exposure to PM_2.5_ indicated in numbers per area of 10 km × 10 km. Areas with the highest mortality are found in South and East Asia, particularly India and China, followed by parts of Africa and Southeast Asia. Densely populated regions show the most significant burden, highlighting stark global health inequalities and the urgent need for air quality improvements in low- and middle-income countries. (*B*) Regional variations in mortality reduction (in %) under different air pollution mitigation strategies, with the highest mortality reductions from emission controls in Central-Eastern Europe, Central Asia, and high-income regions. Southeast Asia and South Asia also show significant reductions. Relatively lower impacts on excess mortality are found in Sub-Saharan Africa due to its lower fossil fuel pollution exposure and the larger role of communicable diseases. These findings emphasize the critical role of fossil fuel reduction in mitigating air pollution-related mortality worldwide (*A* and *B* reproduced from^9^ with permission). In figures *A* and *B*, we present results for both PM2.5 and O_3_ (added). However, since PM2.5 accounts for about 95% of the mortality burden, the figures may also be considered approximately representative for the effects of PM2.5.

Applying the FUSION risk model and consistent with this assessment, Lelieveld *et al*.^9^ estimated the global number of excess deaths from PM_2.5_ and O_3_ at 8.3 (95% CI: 5.6 to 11.2) million per year (*Figure 2*).

About 57% of the global disease-specific excess mortality is attributed to CVD, i.e. IHD and strokes. In Central and Western Europe, with a population of about 550 million, the number of annual excess deaths attributed to air pollution is estimated at 423 000 (292 000 to 550 000).^42^ The excess mortality fraction from cardiovascular relative to other disease-specific mortality in Europe is close to 60%. The global total contribution from exposure to fossil fuel-related air pollution to excess mortality is 61%, i.e. 5.1 (95% CI: 3.6 to 6.3) million deaths annually.^9^ Therefore, a complete theoretical phaseout of fossil fuels would avert 82% of all avoidable deaths from anthropogenic air pollution. Smaller reductions in fossil fuel-related emissions, rather than a radical phaseout, still yield significant positive health outcomes. Different from earlier assessments, it was found that the health benefits respond relatively linearly to the lowering of exposure, suggesting mitigation interventions at all ambient air pollution levels directly translate into health improvement (*Figure 2*). In high-income nations, the halfway scenario (50% reduction of fossil fuel-related emissions) is comparatively most effective because, in some countries, the counterfactual PM_2.5_ level (∼5 µg/m^3^) can be reached under this scenario, effectively reducing mortality to zero. Additional epidemiological analyses are needed to determine if health impacts at PM_2.5_ levels below 5 µg/m^3^ persist. Nevertheless, the 50% phaseout already dramatically improves air quality in all regions, strongly motivating strict ambient air quality legislation, implementation and enforcement, which should be considered a significant and achievable health improvement intervention.

A recent analysis^43^ has addressed general questions about mortality risk calculations. This review showed that disease burden analyses, based on growing epidemiological data and supported by numerous clinical and toxicological studies, have become increasingly robust in recent decades. Concerns about accuracy that were raised several decades ago, such as the representativeness of relatively small cohort studies, have been largely overcome. Nevertheless, challenges persist in fully accounting for confounding factors.

Globally, the health burden of air pollution surpasses the combined mortality from HIV/AIDS, tuberculosis, and malaria, while also resulting in trillions of dollars in annual monetary losses.^44^ Additionally, higher concentrations of air pollution and specific pollutants, such as diesel exhaust (a major source of harmful UFPs in urban environments), have been correlated with increased COVID-19 prevalence and mortality rates, indicating comorbidities related to air pollution, exacerbations and potential synergistic effects.^45,46^ Conversely, lockdowns in many high- and middle-income countries during the COVID-19 pandemic led to reduced air pollution levels, which were associated with decreased cardiovascular events.^47,48^

### 2.3 Epidemiology, air pollution and CVD

In 2019, PM was responsible for 26% of age-standardized CVD deaths in the Eastern Mediterranean region, with CAD being the primary contributor.^49^ Even in high-income countries with relatively low ambient PM levels, long-term exposure remains associated with increased CVD mortality.^50^

Numerous observational studies have consistently linked PM_2.5_ exposure to subclinical atherosclerosis, elevated coronary artery calcium scores, formation of high-risk plaques, and accelerated plaque progression.^51–53^ Long-term exposure is also related to increased carotid intima-media thickness—an established marker of subclinical atherosclerosis—and abnormalities in coronary vasomotor function.^54^ A meta-analysis of 11 European cohort studies demonstrated that each 5 μg/m^3^ increase in annual mean PM_2.5_ was associated with a 13% rise in acute coronary syndrome (ACS) events, while a 10 μg/m^3^ increase in PM_10_ correlated with a 12% increase in ACS events.^55^ More recent large population data on long-term PM2.5 associated ischaemic heart events support these previous findings.^56,57^ Short-term PM exposure has also been associated with a higher incidence of acute myocardial infarction (MI), particularly ST-segment elevation MI, and increased mortality—especially among older individuals with preexisting CAD or major cardiovascular risk factors.^58,59^

Beyond CAD, there is growing consensus on the association between PM exposure and stroke. The GBD 2019 analysis reported that ambient PM_2.5_ was responsible for approximately 1.14 million stroke-related deaths globally.^60^ Supporting this, a recent study in women found that individuals in the highest quartile of PM_2.5_ exposure had a hazard ratio (HR) of 2.14 (95% CI: 1.87–2.44) for all cerebrovascular events compared with those in the lowest quartile.^61^ These female data are complemented by general population data reporting a HR of 1.19 (95% CI: 0.88–1.62), which was further increased in subjects >60 years to 1.40 (95% CI: 1.05–1.87) per annual increment of 5 µg/m^3^.^62^ Both short- and long-term PM exposure have also been linked to increased risk of heart failure (HF), including higher rates of hospitalisation and mortality. A meta-analysis of 35 studies showed that every 10 μg/m^3^ increase in PM_2.5_ and PM_10_ was associated with a 2.12% and 1.63% increase in HF-related hospitalisations and deaths, respectively.^63^ Notably, even in low-pollution regions such as Tasmania, acute PM exposure was associated with higher HF incidence.^53^ PM exposure is also implicated in the development of cardiac arrhythmias, particularly atrial fibrillation (AF). Research involving patients with implantable cardioverter defibrillators revealed that elevated concentrations of PM_2.5_ and PM_10_ were linked to an increased risk of AF and ventricular arrhythmias.^50,64^ A large-scale South Korean study further confirmed that long-term PM exposure was strongly associated with various arrhythmias, with risk increasing proportionally to PM_10_ and PM_2.5_ levels.^65^ A publication from 2025 estimated a yearly global CVD incidence of 5.6 (95% CI: 1.1–9.3) million, attributable to the exposure to UFPs,^66^ using UFP exposure-reponse functions from a Dutch cohort.^67^

There is also robust evidence that PM exposure contributes to the development of cardiovascular risk factors, including hypertension, hyperlipidaemia and diabetes mellitus^68^ and obesity.^69^ A recent meta-analysis found that a 10 μg/m^3^ increase in PM_2.5_ long-term exposure was linked to rises of 0.63 mmHg in systolic and 0.31 mmHg in diastolic blood pressure.^70^ Supporting these findings, randomized trials comparing air filtration to sham filtration revealed that personal air purifiers significantly reduced mean systolic blood pressure by nearly four mmHg (95% CI: –7.00 to –0.89) over a median duration of 13.5 days.^71^ Studies have demonstrated significant associations between PM exposure and elevated levels of total cholesterol, triglycerides, and low-density lipoprotein (LDL) cholesterol.^72^ In the GBD 2019 analysis, PM_2.5_ was identified as the third leading environmental risk factor for diabetes, accounting for roughly one-fifth of the global diabetes burden and contributing to approximately 13.4% of diabetes-related deaths.^73^

### 2.4 Pathophysiology of air pollution-induced CVD

This section outlines the mechanistic pathways through which air pollution promotes the development and progression of atherosclerosis, the major pathology underlying many CVD, drawing primarily from recent *in vitro* and *in vivo* experimental evidence.

Adverse cardiovascular effects are, to some extent, consistent across various particles and reactive gases, including PM_2.5_ and UFPs.^74–77^ Exposure to PM_2.5_ increases circulating sphingolipids—bioactive lipids that stimulate the production of apolipoprotein B-containing lipoproteins, which are causally linked to atherogenesis and cardiovascular risk.^78,79^ While LDL cholesterol initiates plaque formation, high-density lipoprotein (HDL) protects against atherosclerosis via reverse cholesterol transport and anti-inflammatory functions. However, air pollution impairs HDL functionality by promoting oxidative modifications and reducing apolipoprotein A-I levels, thus attenuating cholesterol efflux.^80^

Oxidative stress is a key pathogenic mechanism. Both PM_2.5_ and UFPs generate ROS, reducing endogenous nitric oxide bioavailability, and disrupting endothelial function.^25^ These effects are potentiated by surface-bound constituents in PM such as heavy metals and PAHs.^81^ The systemic consequences extend beyond the vasculature, inducing cerebrovascular damage and neuroinflammation. In ApoE^−/−^ mice, a common model for athersoclerosis, PM_2.5_ exposure upregulates oxidative stress markers and activates the Nrf2 antioxidant defence pathway.^82^ These oxidative conditions facilitate LDL oxidation, promoting the formation of oxLDL, which is avidly taken up by macrophages to form foam cells—an early feature of atherogenesis.^83^ Diesel exhaust particles (DEP) and other traffic-related emissions impair HDL antioxidant capacity and increase systemic lipid peroxidation (for review see^84^). Even in the absence of PM, gaseous emissions from gasoline engines elevate vascular oxidative stress and endothelin-1 levels, promoting vasoconstriction.^85^ These changes contribute to myocardial ischaemia, diastolic dysfunction, and reduced cardiac contractile reserve, especially in vulnerable populations.^86^

Inflammatory pathways are also a hallmark of air pollution exposure. Exposure to PM_2.5_ increases levels of pro-inflammatory cytokines such as tumour necrosis factor alpha (TNF-α), monocyte chemoattractant protein-1 (MCP-1), and interleukin-12 (IL-12), while suppressing anti-inflammatory IL-10.^87^ The nicotinamide adenine dinucleotide phosphate oxidase (NADPH) oxidase (NOX2)-mediated Toll-like receptor signalling enhances oxidative stress and induces inflammatory lipid mediators, including 7-ketocholesterol and oxidized phospholipids (oxPAPC).^88^

PM_10_ elevates IL-6 levels and promotes endothelial expression of ICAM-1 and VCAM-1, facilitating monocyte adhesion and transmigration.^89^ Due to their small size and high surface area, UFPs translocate across the alveolar epithelium, enter the bloodstream, and accumulate in atherosclerotic plaques.^90^ Furthermore, inhaled UFPs can penetrate remote organs such as the heart and brain, where they elicit oxidative and inflammatory responses.^25^ These particles can also activate the sympathetic nervous system via pulmonary afferents or olfactory nerve pathways, contributing to hypertension, MI, and neuroinflammation.^91–93^ In contrast, larger microparticles predominantly remain in the lungs, causing localized pulmonary inflammation.^66^ DEP and ozone exacerbate endothelial dysfunction via CD36, which mediates oxLDL uptake and foam cell formation.^94^ PM exposure increases circulating monocyte levels, partially driven by cytokine release from lung-resident immune cells.^95^ Emerging data suggest that local vascular oxidative injury may be more relevant than systemic inflammation in the pathogenesis of atherosclerosis.^96^

Foam cell accumulation in atherosclerotic plaques is aggravated by increased CD36 expression, enhanced lipid uptake, mitochondrial damage, and defective efferocytosis due to tyrosine kinase MerTK downregulation, ultimately promoting necrotic core expansion.^97,98^ These processes are strongly influenced by exposure to PM2.5, especially from traffic-related air pollution. For example, PM2.5 can polarize macrophages toward a pro-inflammatory M1 phenotype and impair their ability to clear apoptotic cells, a key step in plaque resolution.^97,98^

Additionally, T-cell activation, particularly of Chemokine receptor type 3 (CXCR3^+^) CD4^+^ and CD8^+^ subsets, drives a helper T-cell (Th1)-skewed immune response. This favours the activation of the NOD-like receptor protein 3 (NLRP3) inflammasome and subsequent pro-inflammatory cytokine release, contributing to plaque instability and potential rupture.^99,100^ Notably, DEP can amplify this immune activation through non-canonical IL-1β pathways, further aggravating vascular inflammation.^100^ In addition, IL-1β and IL-6 are key pro-inflammatory cytokines released by alveolar macrophages following the exposure to PM2.5 are contributing to CVD and its risk factors.^101–103^

As plaques evolve, thin fibrous caps form over necrotic cores predisposes to rupture.^104^ PM enhances the expression of matrix metalloproteinases (MMP-2 and MMP-9), impairs extracellular matrix stability, and increases necrotic burden, all contributing to plaque destabilisation.^105,106^ In ApoE^−/−^ mice exposed to DEP morphological changes in plaques suggest the presence of previous plaque ruptures.^85^ In both animal and human studies, PM_2.5_ exposure has been associated with platelet activation and elevated thrombogenic mediators such as CD40 ligand and fibrin degradation products.^107–109^

Beyond classical vascular injury, air pollution interferes with circadian rhythm regulation. PM_2.5_ disrupts core clock genes such as *BMAL1* and *CLOCK*, producing effects similar to those of nocturnal noise and artificial light exposure (see below).^110,111^ Circadian disruption is a recognized cardiovascular risk factor for metabolic dysregulation, insulin resistance, and obesity.^112,113^ Recent experimental models of combined exposure to PM and aircraft noise reveal synergistic cardiovascular toxicity: PM primarily induces oxidative stress and pulmonary inflammation, whereas noise activates neuronal and systemic stress responses, both converging to amplify cardiovascular damage.^91,114^ In summary, air pollution promotes atherogenesis and cardiovascular events through converging oxidative stress, inflammation, immune dysregulation, endothelial dysfunction, circadian misalignment, and plaque destabilisation—mechanisms.

## 3. Noise pollution

**Figure 3:**
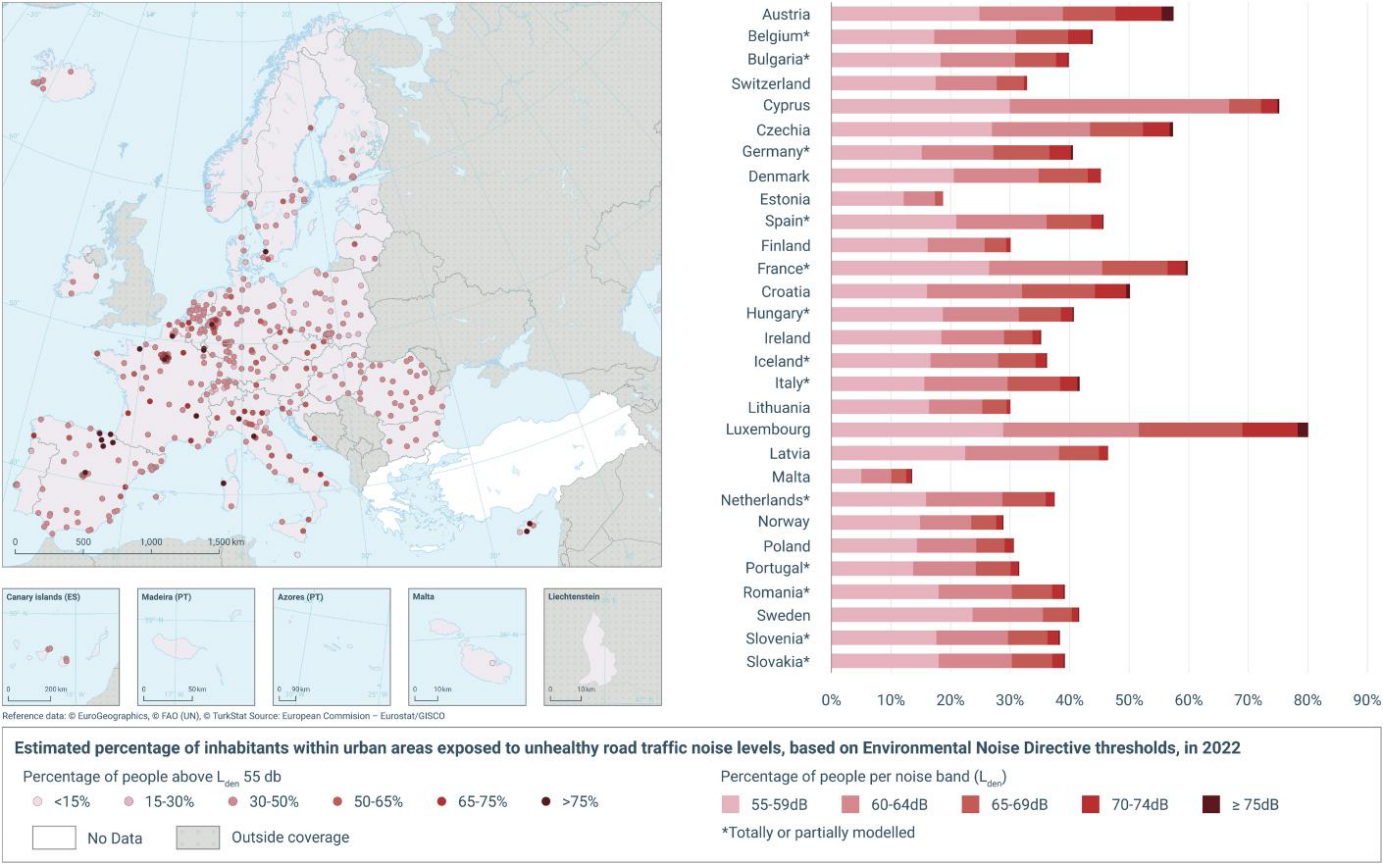
Estimated percentage of residents within larger European cities exposed to road traffic noise of 55 dB or more in 2022. Calculations are performed by all EU member countries as part of the EU Environmental Noise Directive and summarized by the EEA.^115^ Exposure levels vary widely, with Luxembourg, Germany, and Czechia reporting the highest proportions, with >60% exposed in urban areas. In contrast, countries like Estonia and Malta report much lower percent exposed.

Noise is a ubiquitous exposure, especially in urban populations. In a 2022 noise mapping of the European Union, more than 20% of the population was reported to live in areas where transport noise levels exceeded the EU threshold (55 dB Lden noise over a whole day, with a penalty of 5 dB for evening noise and 10 dB for night noise)^115^ (*Figure 3*). When applying the stricter threshold of 53 dB for road traffic noise recommended by the WHO,^116^ this number increased to more than 30%. Importantly, this mapping is mainly based on noise estimation in larger urban areas, and in these areas, most countries reported that 30–60% of the population was exposed to above 55 dB (*Figure 3*).

In 2018, a WHO expert panel conducted a systematic review reporting that road traffic noise is associated with a higher risk of IHD based on high-quality evidence.^116^ This quality assessment was performed using the Grading of Recommendations Assessment, Development and Evaluation, with evaluation of several criteria for each study, including study design, consistency and precision of the results, directness of the evidence, publication bias, and exposure-response gradient. For other CVD, the panel concluded that there was either very low, low, or moderate quality evidence, or that the evidence was not evaluated, e.g. for heart failure. Since then, many studies investigating associations between transport noise and the risk of CVD have been published,^117,118^ necessitating an updated evaluation of evidence, as this information is a vital input in health risk assessments.

In collaboration with Swiss and Spanish researchers, the European Environment Agency (EEA) recently conducted an Umbrella+ review of epidemiological studies on the health effects of environmental noise, with subsequent meta-analyses and evidence evaluation, applying the same criteria as used in the 2018 WHO report.^119^ In this comprehensive evaluation, risk estimates from the 2018 WHO report were updated with those obtained in more recent studies (published until mid-2023), mainly identified using high-quality systematic reviews. The Umbrella+ review concluded that long-term exposure to road traffic noise was associated with a higher risk of IHD based on high-quality evidence, with an estimated relative risk (RR) of 1.04 (95% CI: 1.02–1.06) per 10 dB higher noise. The Umbrella+ review also concluded that road traffic noise is associated with a higher risk of incident stroke (RR: 1.05, 95% CI: 1.01–1.08 per 10 dB) and heart failure (RR: 1.04, 95% CI: 1.02–1.07 per 10 dB) as well as cardiovascular mortality (RR: 1.05, 95% CI: 1.02–1.07 per 10 dB). For arrhythmias, only few cohort studies exist, and the quality evidence was concluded to be moderate with an RR of 1.01 (1.00–1.02) per 10 dB road traffic noise. Although many studies have investigated associations between transport noise and hypertension, these are mainly of cross-sectional design, and the quality of evidence was evaluated to be low. For all CVD, the proof of an association with airport and railway noise remains limited and inconsistent,^119^ highlighting the need for more studies on the health impacts of these exposures.

The threshold at which transport noise no longer affects cardiovascular health has yet to be determined. Currently, the EU calculates transport noise and health impacts from 55 dB Lden and up, while the WHO 2018 recommended a threshold of 53 dB for road traffic noise and 45 dB for aircraft noise to protect the population.^116^ Recent studies on large populations with substantial variations in noise exposure (from 35 to 40 dB and up) have shown associations between road traffic noise and CVD at levels below 53 dB.^117,118^ Based on this, the Umbrella+ review recommended assessment of the health risks from noise levels of 45 dB Lden and up.^119^

In addition to the established associations between chronic exposure to road traffic noise and CVD described above, recent studies have investigated whether short-term noise can trigger CVD. A study from Switzerland found that nighttime aircraft noise of 40–50 dB and >50 dB within two hours before CVD death was associated with odds ratios of 1.33 (1.05–1.67) and 1.44 (1.03–2.04), respectively.^120^ A study based on the population living near Heathrow Airport (London, UK) found small associations between levels of evening aircraft noise and cardiovascular hospitalisations, but no associations with CVD death or with other exposure periods.^121^

Several studies have examined the associations between transport noise and key cardiovascular risk factors and comorbidities, including metabolic disease and poor mental health.^119,122–124^ These studies have consistently shown road traffic noise is associated with a higher risk of type 2 diabetes, resulting in an evaluation of high quality evidence in the recent EEA report with an estimated pooled RR of 1.06 (1.03–1.09) per 10 dB road traffic noise.^119^ Similarly, road traffic noise has been associated with adiposity measures and mental health outcomes, including depression, anxiety and suicide^119,123,124^ suggesting that these are important contributors on the pathway between noise and CVD.

Health effects of transport noise among children and adolescents were evaluated in a recent Umbrella+ review from the EEA.^125^ For these age groups, most previous studies focused on noise effects on reading and oral comprehension, behavioural problems, and being overweight, based on which the review concluded moderate certainty of evidence for an association with noise. The review also identified five papers investigating associations between transport noise (in school, home, or both) and blood pressure in children. The results of these studies are, however, inconsistent, and more research is needed to determine whether noise increases cardiovascular risk markers in childhood.

### 3.1 Translational studies explaining the noise-induced pathophysiology

#### 3.1.1 Translational noise studies in humans

Field studies show that nighttime aircraft and railway noise negatively impact vascular function, sleep quality, and stress-related biomarkers in both healthy individuals and those with CVD.^126,127^ These effects appear to be dose-related, becoming more severe at higher levels of exposure. Exposure to aircraft noise (30–60 events per night) at a sound level (Leq) of 46.3 dB(A) with peaks at 60 dB(A) led to endothelial dysfunction and increased adrenaline levels, impairing vascular function measured by flow-mediated dilation (FMD).^127^ The effects were worse with prior noise exposure, indicating a priming effect. Vitamin C supplementation improved endothelial function, suggesting ROS drive noise-induced vascular damage.^128^ Increased oxidative stress markers, such as 3-nitrotyrosine and 8-isoprostane, further confirmed this hypothesis. Railway noise had similar effects, impairing FMD and elevating oxidative stress and inflammatory biomarkers.^129^ Importantly, the worsening of endothelial function in response to noise was more pronounced in patients with established CVD compared with healthy subjects.^130^ Studies have found that both infrequent loud and frequent lower-level aircraft noise at night caused endothelial dysfunction and diastolic heart dysfunction.^131^ Long-term noise exposure alters immune function, increasing levels of interleukin-12 and high-sensitivity C-reactive protein, while reducing natural killer cell activity.^132,133^ The Swiss SAPALDIA cohort identified DNA methylation changes affecting inflammation pathways in individuals exposed to chronic noise.^134^ Additionally, long-term exposure to train or road traffic noise was linked to arterial stiffness and early-stage atherosclerosis,^135,136^ further strengthening the close link between noise and CVD.

#### 3.1.2 Translational noise studies in animals

Animal studies have also reveal that noise exposure triggers vascular dysfunction. Mice exposed to aircraft noise (Leq 72 dB(A)) exhibited increased stress hormones, elevated blood pressure, and ROS generation through NADPH oxidase (NOX-2) activation.^137^ Noise also uncoupled endothelial nitric oxide synthase, reducing nitric oxide bioavailability and impairing vascular function. Importantly, white noise exposure under similar conditions did not cause these effects, highlighting that noise characteristics, not just intensity, are critical.^137^ Further studies showed that noise-induced vascular constriction and inflammation were absent in NOX-2 knockout mice.^128,138^ Inflammatory markers, including interleukins and immune cells, were significantly elevated in noise-exposed mice.^139^ These findings suggest shared mechanisms between noise-induced vascular damage and other CVD risk factors like diabetes and hypertension.^140^ Noise exposure dysregulated genes related to vascular integrity, particularly in pathways associated with TGF-β signalling, autophagy, and inflammation. RNA sequencing in noise-exposed mice revealed disruptions in NF-κB signalling, circadian rhythm, and oxidative stress.^128,137^ Nighttime noise exposure had more severe effects, impairing Foxo3 signalling and exacerbating neuroinflammation. Mice exposed to noise during sleep experienced more significant cardiovascular damage than exposure during wakefulness, emphasising the role of circadian rhythms.^128^ Noise-induced oxidative stress and inflammation affected vascular and cerebrovascular systems, reinforcing the need to mitigate nighttime noise exposure. Chronic noise exposure led to persistent endothelial dysfunction and oxidative stress in mice, with no signs of adaptation over 28 days.^141^ However, after 4 days of noise cessation, endothelial function in large vessels normalized, though some microvascular attenuation remained.^142^ These results indicate that prolonged quiet periods may be essential for full vascular recovery. In a recent study, aircraft noise was found to worsen cardiovascular outcomes in three mouse models of diabetes: type 1 diabetes (streptozotocin-induced), type 2 diabetes (S961 insulin receptor antagonist-induced), and metabolic syndrome (high-fat diet-induced).^143^ Noise exposure exacerbated hyperglycaemia and endothelial dysfunction in all models, leading to increased blood pressure, more pronounced endothelial dysfunction, and increased oxidative stress. Mitochondrial assessments revealed noise-induced impairments in respiratory chain function, further compounding diabetes-related cardiovascular risks. These findings strongly suggest that noise amplifies metabolic and cardiovascular complications in diabetics.^144^

**Figure 4:**
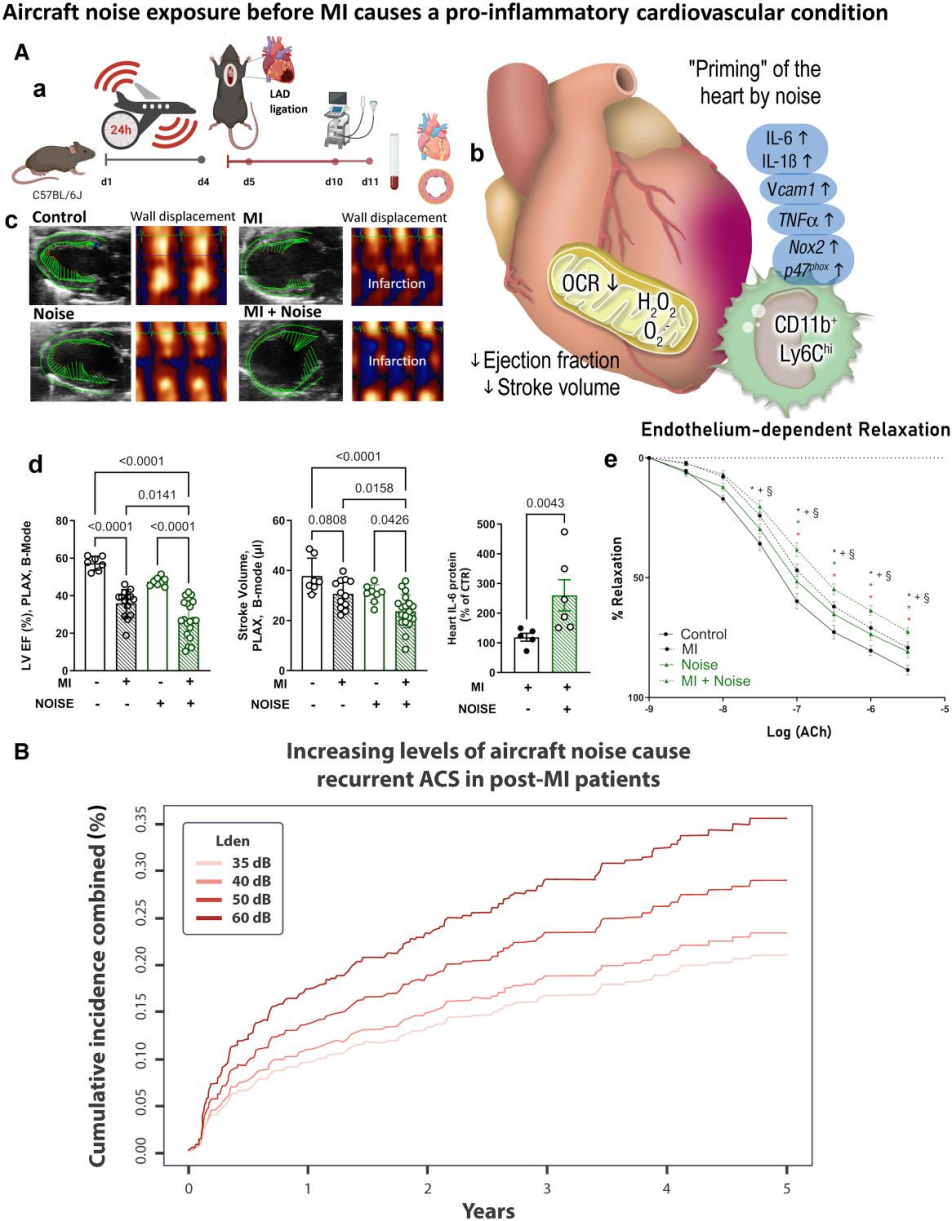
The impact of aircraft noise exposure on cardiovascular health Panel (*A*): An experimental model of MI in mice showing that (a) prior noise exposure worsens cardiac function post-MI. (b) noise-induced “priming” of the heart, leading to increased inflammation, oxidative stress, and endothelial dysfunction. (c) echocardiographic images showing worsened infarction with noise exposure. (d) and (e) statistical data demonstrating reduced ejection fraction, stroke volume, and endothelial relaxation with noise. Panel *B*: A dose-dependent relationship between noise levels and recurrent ACS in post-MI patients. (*A*): with permission from^148^; (*B*): with permission from.^149^ C57BL/6J, ‘black six’ mouse strain; LAD, left anterior descending artery; OCR, oxygen consumption rate; IL, interleukin; Vcam1, vascular cell adhesion protein 1; TNFα, tumour necrosis factor alpha; Nox2, catalytic subunit of the phagocytic NADPH oxidase (gp91phox); p47phox, regulatory cytosolic subunit of the phagocytic NADPH oxidase; CD11b, integrin α-M (Mac-1); Ly6C, lymphocyte antigen 6 (uPAR); LVEF, left ventricular ejection fraction; PLAX, parasternal long axis view; ACh, acetylcholine; L_den_, day–evening–night noise level (weighted noise average over an entire day); dB, decibel.

Noise activates stress pathways involving the renin-angiotensin-aldosterone system and sympathetic nervous system, mediated by oxidative stress and NOX-2 activation.^137,145^ Noise exposure increased levels of angiotensin-II, endothelin-1, and catecholamines, intensifying vascular inflammation and oxidative stress.^137^ Similar mechanisms are seen in classical hypertension models.^146^ Human studies highlight the amygdala's role in linking noise to stress-induced vascular inflammation and increased cardiovascular risk.^147^ Noise exposure before acute stressors worsened MI outcomes in mice, increasing infarct size, oxidative stress, and cardiac dysfunction^148^ (*Figure 4*).

The findings of some of the preclinical studies in mice have been reproduced in humans. Individuals exposed to high levels of aircraft noise had poorer MI prognosis, with higher inflammatory markers and reduced left ventricular ejection fraction.^148^ Furthermore, clinical studies in patients following ACS found that aircraft noise increased the risk of recurrent cardiovascular events in patients with ACS^149^ (*Figure 4*).

Non-pharmacological interventions such as physical activity, intermittent fasting, and pharmacological activation of endothelial AMP-kinase (AMPK) mitigated noise-induced vascular damage in mice.^144^ These approaches also restored endothelial function and reduced oxidative stress. In endothelial-specific AMPK knockout mice, these protective effects were absent, highlighting the critical role of the endothelial AMPK's in noise resilience. This suggests that lifestyle modifications and pharmacological AMPK activation could serve as effective countermeasures against noise-induced cardiovascular dysfunction.

We recently studied the protective effects of cardiovascular drugs against aircraft noise-induced vascular damage using an established mouse model. Mice were exposed to aircraft noise (72 dB(A)) for 4 days while treated with the beta-blocker propranolol or the alpha-blocker phenoxybenzamine.^150^ Noise exposure caused hypertension and impaired endothelial function in large arteries and cerebral microcirculation, accompanied by increased oxidative stress and inflammation. Treatment with propranolol and phenoxybenzamine effectively preserved endothelial function. It reduced oxidative stress and inflammation in heart tissue, suggesting that pharmacological dampening of the sympathetic nervous system may represent a practical approach to ameliorate cardiovascular side effects of noise.^150^

## 4. Outdoor light pollution

Light pollution is a growing environmental concern, affecting approximately 83% of the global population and nearly all individuals in the USA and EU.^151^ Exposure to artificial light at night disrupts circadian rhythms, increased premature mortality.^152^ In humans, circadian misalignment contributes to CVD by impairing inflammatory control in atherosclerosis^113^ and altering metabolic pathways linked to obesity and hyperglycaemia.^153,154^ Individual chronotypes, determined by genetic predisposition to ‘morningness’ or ‘eveningness’, influence diabetes risk and overall health outcomes.^155,156^ Studies associate artificial light at night with an elevated risk of coronary heart disease (CHD) and mortality in older adults.^157^ Higher exposure levels correlate with increased CHD hospitalisations and mortality, particularly when combined with air pollution, in both human and animal studies.^110,158^ Overweight or obese individuals appear more susceptible to these adverse effects, emphasising the interaction between environmental and metabolic health factors. Animal models provide further evidence of the impact of disrupted light cycles on cardiovascular health. In a shift work model, light-dark cycle alterations led to higher stroke-induced mortality in male rats, while female rats experienced greater infarct volumes and sensorimotor deficits.^159^ Constant light exposure in high-fat-fed rats exacerbated glucose abnormalities, insulin resistance, and inflammation leading to liver disease.^160^ Similarly, chronic circadian disruption increased atherosclerosis and dyslipidemia in female, but not male, ApoE^−/−^ mice.^161^ Light pollution also influences cardiovascular function in humans. A 5 lux increase in outdoor night-time lighting was associated with a 3–4 mmHg rise in blood pressure among elderly individuals.^162^ A meta-analysis further linked night-time light exposure to elevated risks of HF, CHD, stroke, and MI in a study of 579 Chinese counties.^163^ More extensive research is required to validate these findings across diverse populations and age groups.

## 5. Climate change and extreme temperature, desert storms, and wildfires

### 5.1 Extreme temperatures or non-optimal temperatures

High air temperatures pose significant health risks, whether occurring during isolated hot days or prolonged heatwaves. These dangers extend beyond immediate effects like dehydration or heatstroke to exacerbating chronic conditions, including CVD, respiratory illnesses, kidney disorders, and electrolyte imbalances.^164^ Individuals with pre-existing health issues, particularly those with CVD, are especially vulnerable, leading to increased emergency room visits and hospital admissions.^165–167^ Several factors, including age, socioeconomic status, and underlying health conditions, heighten the risk of heat-related acute cardiovascular events such as MI and acute left heart decompensation.^168^ Furthermore, environmental conditions, particularly air pollution, can compound the health effects of high temperatures, worsening health outcomes.^169^ Rapid urbanisation, an aging population, and shifting socioeconomic development pathways also amplify vulnerability to heat stress.^170^ With global climate change continuing to worsen, the frequency, duration, and intensity of heatwaves are expected to rise.^171^

The 2019 GBD study identified non-optimal temperatures (NOT) (heat or cold) as a significant risk factor for human health, contributing to the loss of 11.7 million DALYs globally.^172^ According to the WHO, climate change could lead to 250 000 deaths annually between 2030 and 2050 due to increased heat exposure, particularly among the elderly, and rising incidences of diarrheal diseases, malaria, dengue, and childhood stunting.^173^ These figures are likely underestimations of the full burden of climate-change-related mortality as they exclude other climate-sensitive health conditions and extreme weather's effects on health services, as well as the indirect effects of climate change in food systems, availability of clean water, sanitation, social economic insecurity, and population displacement.

Extreme temperatures contribute significantly to cardiovascular morbidity and mortality.^174,175^ A 2021 global analysis estimated that over 5 million deaths annually are linked to NOT.^176^ Although the relationship between outdoor air temperature and cardiovascular mortality appears already very robust, the effects of temperature on cardiovascular morbidity are smaller and more variable.^177^ Cold-related deaths currently outnumber heat-related ones, but increasing heatwaves are shifting this balance.^178^ The human body responds to heat stress by redistributing blood flow and secreting sweat, which, in individuals with compromised cardiovascular function, can result in ischaemia, infarction, and cardiovascular collapse.^179,180^ Cardiovascular strain from heat stress, particularly among older adults, is a leading cause of heatwave-related mortality.^170^

#### 5.1.1 Interaction of heat with air pollution

**Figure 5:**
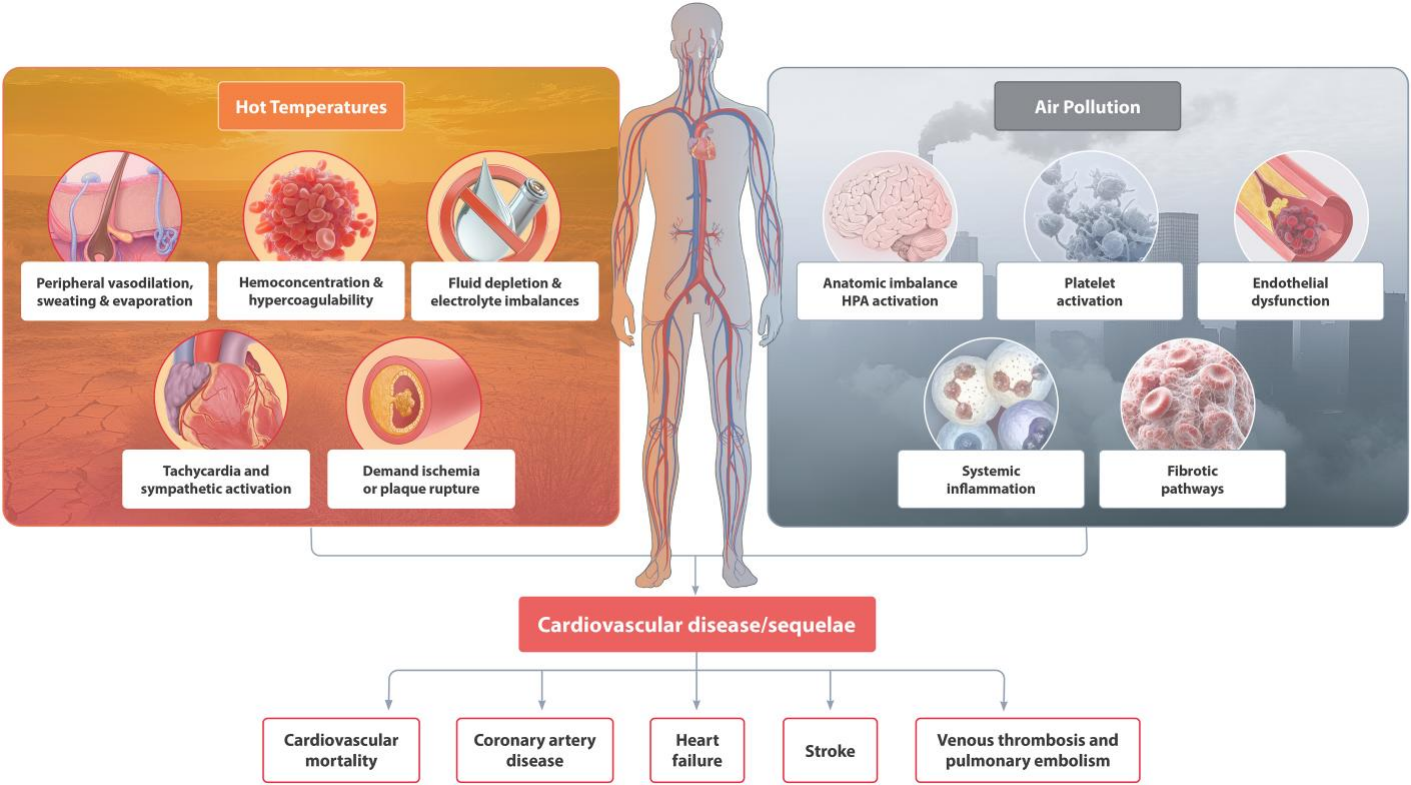
The impact of extreme heat and air pollution on cardiovascular health. High temperatures cause vasodilation, dehydration, electrolyte imbalances, hypercoagulability, and increased cardiac strain, potentially leading to ischaemia or plaque rupture. Air pollution contributes to endothelial dysfunction, platelet activation, systemic inflammation, and fibrotic changes, further exacerbating cardiovascular risk. These combined stressors increase the likelihood of CAD, heart failure, stroke, venous thrombosis, and cardiovascular mortality. The interaction between heat and pollution underscores the urgent need for mitigation strategies to protect cardiovascular health in an increasingly warming and polluted environment. Modified from^183^ with permission.

Interactions between rising temperatures and air pollution magnify cardiovascular risk. High temperatures can coincide with stagnant atmospheric conditions, promoting the photochemical formation of air pollutants and preventing their dispersion. Temperature inversions can similarly trap pollutants, leading to episodes of extreme cold and increased air pollution, heightening cardiovascular risk. Epidemiological studies have demonstrated that high temperatures and air pollution collectively increase CVD mortality. Research has shown that PM_2.5_ exacerbates the association between rising temperatures and CVD mortality, with the combined effect being more significant than the impact of each factor alone^181,182^ (*Figure 5*). The two environmental factors share common pathomechanisms in many regulatory processes in the body. It is therefore conceivable that interactions and synergies between air temperature and air pollutants are likely.^184^ However, studies from California have reported inconsistencies in the interactive effects of extreme PM_2.5_ and heat.^185^ Yet a global analysis of 482 cities found that pollutants such as PM_10_, PM_2.5_, O_3_ and NO_2_ amplified high temperatures’ effects on CVD mortality, with O_3_ and NO_2_ showing the most pronounced impact Collectively, these findings emphasize the need to consider air pollution and temperature as interconnected factors influencing cardiovascular health.

### 5.2 Desert dust

Airborne soil contamination is an often-overlooked health risk. Agricultural activities, unpaved roads, and construction contribute to dust emissions, but the largest source is desert wind erosion, particularly from the ‘dust belt’ spanning North Africa, the Middle East, and parts of Asia.^186^ Desert dust can account for 30–50% of atmospheric aerosols^187^ and can travel vast distances, affecting populations far from its origin. Desert dust is not entirely natural—anthropogenic influences, such as industrial pollution, exacerbate its toxicity.^188^ Cardiopulmonary mortality linked to desert dust exposure is estimated at 1.8% globally (actually up to 0.99 million deaths per year) but reaches 15–50% in highly affected regions.^189^ As urban and industrial air pollution declines due to regulatory efforts, climate change is projected to make desert dust a dominant air quality concern, especially due the drying effects of temperatures increasing the potential aerosolization of ground dust.^190^ Desert dust exposure induces oxidative stress, inflammation, and respiratory tract damage.^191^ Fine dust particles provoke systemic responses, impacting cardiovascular and immune functions.^192^ Toxicity increases when desert dust interacts with urban pollutants, forming sulfates and nitrates (contaminated with toxic metals and polycyclic hydrocarbons) that enhance oxidative stress.^193^ Studies in China confirm that desert dust passing through industrial areas carries higher levels of pollutants, amplifying its harmful effects.^194^ Epidemiological research has linked desert dust exposure to cardiovascular mortality. In Japan, Asian dust events were associated with an increase in acute MI.^195^ A meta-analysis found that each 10 µg/m^3^ increase in PM_10_ dust exposure correlates with a 2% rise in cardiovascular mortality, persisting for up to two days post-exposure.^196^ Further research is needed to assess the long-term cardiovascular consequences of chronic desert dust exposure, and to what extent the dust itself engenders risk compared with the other sources of constituents it may carry.

### 5.3 Wildfires

**Figure 6:**
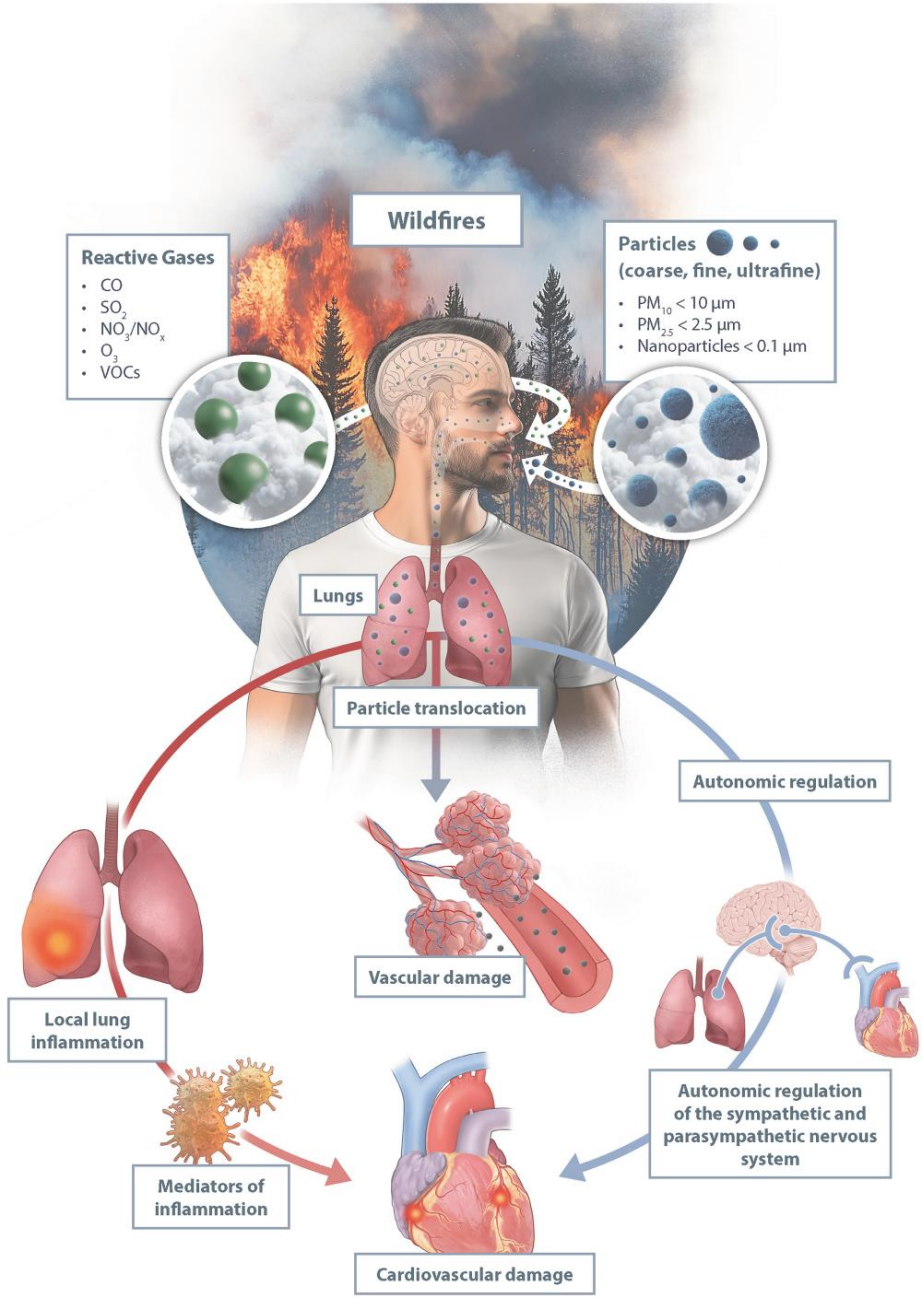
This figure illustrates the impact of wildfire smoke on cardiovascular health. Wildfires release reactive gases (CO, SO_2_, NO_x_, O_3_, volatile organic compounds (VOCs)) and particulate matter (PM_10_, PM_2.5_, ultrafine particles), which enter the lungs via inhalation. Fine particles can translocate into the bloodstream, causing vascular damage, inflammation, and oxidative stress. Additionally, inhaled pollutants disrupt autonomic nervous system regulation, contributing to cardiovascular dysfunction. Local lung inflammation triggers systemic inflammation, further exacerbating heart and vascular damage. These combined effects increase the risk of CVD such as heart attacks and strokes.

Climate change has intensified wildfire frequency and severity. Large wildfires have occurred in Greece, Australia, Brazil, and the U.S., where over 70 000 wildfires occur annually, and the burned acreage has tripled over the past 30 years.^197^ Between 2008 and 2012, more than 10 million individuals were exposed to hazardous air pollution from wildfires for extended periods.^198^ Wildfire smoke can travel thousands of kilometres, as demonstrated by the June 2023 Canadian fires, which affected air quality across major U.S. cities, such as New York. Wildfires are a significant source of air pollution, emitting PM_2.5_, toxic gases, and volatile organic compounds. In 2005 wildfires contributed ∼18% of U.S. PM_2.5_ emissions,^199^ and exposure has increased by 77% since 2002.^200^ PM_2.5_ levels during wildfires often exceed 300–500 µg/m^3^, rivalling the pollution levels of the world's most contaminated megacities.^201^ The toxicity of wildfire PM varies depending on biomass composition, burning conditions, and the combustion of other material that may have been present in the blaze.^202^ Some studies have suggested wildfire PM is more harmful than urban PM due to smaller particle sizes compared with some other sources of urban PM, oxidative potential, and co-exposure to extreme heat,^201^ although it is difficult to address other confounding influences (*Figure 6*). Wildfire smoke is responsible for an estimated 339 000 to 675 000 premature deaths annually.^203^ The 2023 Canadian wildfires were linked to increased hospital admissions for respiratory and cardiovascular conditions in the U.S.^204^ Meta-analyses indicate that every 10 µg/m^3^ increase in wildfire PM_2.5_ is associated with a 1.9–3.3% rise in cardiovascular mortality.^205^ Wildfire smoke exposure is linked to increased hospitalisations for acute coronary events, stroke, cardiac arrhythmias, HF exacerbation, and hypertensive crises^197,206^ (*Figure 6*). Firefighters exposed to wildfire smoke have demonstrated increased arterial stiffness, elevated inflammatory markers, and impaired vascular function.^207^ Experimental studies reinforce these findings. Controlled exposure to woodsmoke in humans raises blood pressure, impairs vascular function, and promotes coagulation.^208^ Long-term indoor biomass burning is linked to carotid atherosclerosis.^209^ In preclinical models, wildfire PM induces oxidative stress, DNA damage, and ischaemic cardiac injury.^210,211^ Mitigation strategies are crucial for reducing wildfire-related cardiovascular risks. Forecasting high-risk events, educating citizens and patients, and adjusting medication regimens during smoke episodes can minimize health impacts.^212^ Given the increasing prevalence of wildfires, further research is needed to understand their long-term cardiovascular effects fully, and develop advice for those most at risk.

## 6. Chemical pollution and plastics

Contaminated soil and water significantly threaten human health through exposure to toxic chemicals. According to the WHO, 2 million deaths and 53 million DALYs were lost in 2019 due to chemical exposures, a sharp rise from 2016 figures (1.6 million deaths and 45 million DALYs).^213^ Hazardous substances include heavy metals, PAHs, per- and polyfluorinated substances (PFAS), pesticides, and organic solvents. While these chemicals have been linked to cancer and respiratory diseases, they are increasingly associated with CVD.^213^ While chemical exposures can be very common in populated regions of LMICs, biomonitoring studies have also detected numerous chemicals in both European and U.S. populations.^214,215^

### 6.1 Cardiovascular effects of chemical pollutants

#### 6.1.1 Heavy metals

**Figure 7:**
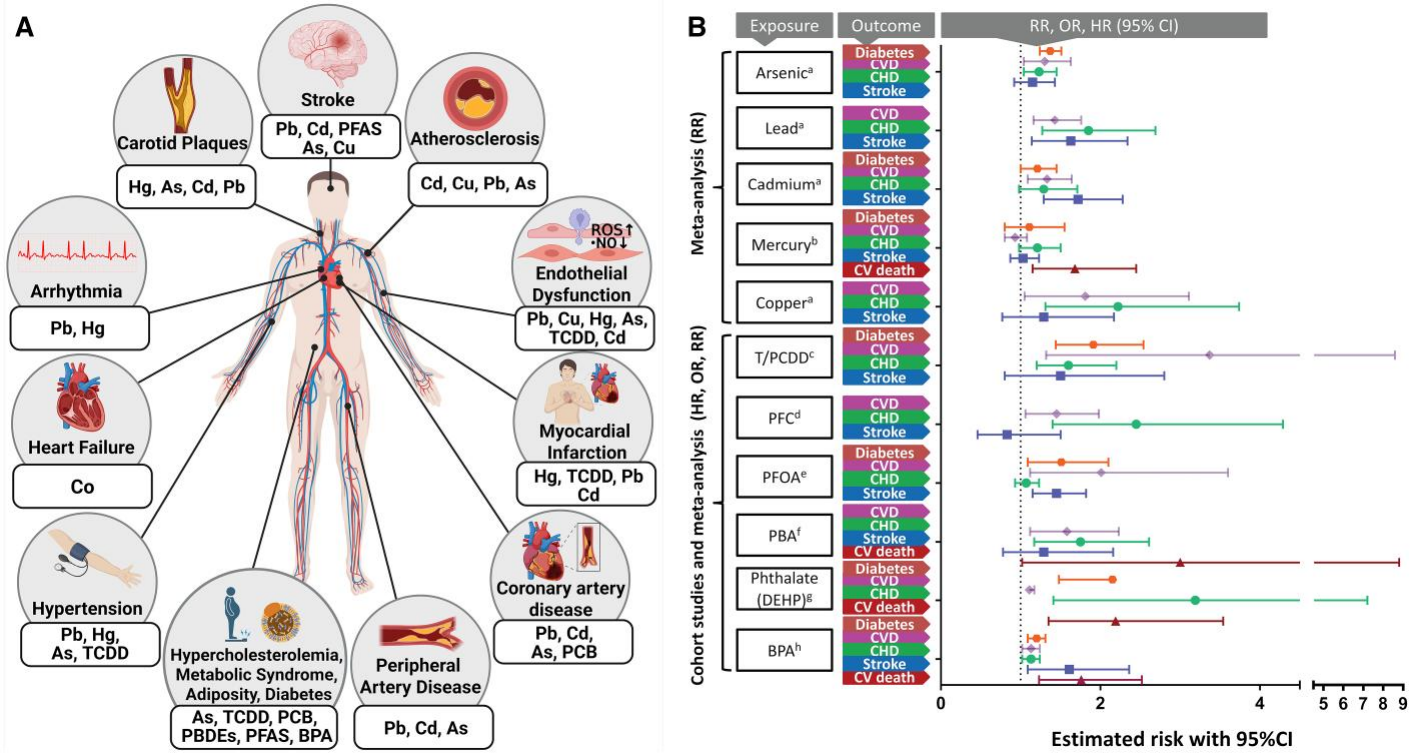
Association between metals, pesticides and cardiovascular and cardiometabolic outcomes. (*A*) Overview of health effects of different toxic chemicals. TCDD, 2,3,7,8-tetrachlorodibenzo-p-dioxin; PFAS, per- and polyfluorinated substances; PCB, polychlorinated biphenyls; PBDE, polybrominated diphenyl ethers; ROS, reactive oxygen species. (*B*) The graph shows the risk estimates for major CVDs, cerebrovascular events and cardiovascular (CV) deaths associated with different chemical pollutants. The data were derived from meta-analyses or cohort studies. BPA, bisphenol A; CHD, coronary heart diseases; DEHP, diethylhexyl phthalates; HR, hazard ratio; OR, odds ratio; PBA, phenoxybenzoic acid; PCDD, polychlorinated dibenzo-p-dioxins; PFC, perfluorinated and polyfluorinated chemicals; PFOA, perfluorooctanoic acid; RR, relative risk. Figure compiled from evidence based on: ^a^ data on arsenic, lead, cadmium and copper taken from.^219,228,229^  ^b^ Data on mercury taken from.^230,231^  ^c^ Data on PCDD taken from.^232–234^  ^d^ Data on PFC taken from.^235–237^  ^e^ Data on PFOA taken from.^238–241^  ^f^ Data on PBA taken from.^242,243^  ^g^ Data on DEHP taken from.^244–247^  ^h^ Data on BPA taken from.^248–252^ Right panel adapted from^81^with permission.

Heavy metals such as arsenic, cadmium, lead, and mercury are major risk factors for CVD. Lead exposure, even at low concentrations, is a well-established cause of hypertension and cardiovascular mortality.^10,216,217^ Cadmium exposure is associated with CAD, atherosclerosis, and HF,^10,218,219^ with oxidative stress, vascular damage, and endothelial dysfunction playing central roles.^220^ Methylmercury contributes to carotid atherosclerosis and MI risk,^221^ while copper promotes atherosclerosis through cuproptosis.^222^ Arsenic exposure has been associated with increased carotid intima-media thickness, a surrogate for early atherosclerosis and IHD.^223,224^ Preclinical studies confirm these effects, with ApoE^−/−^ mice showing increased plaque formation upon arsenic and cadmium exposure.^225,226^ Mercury exposure similarly exacerbates atherosclerosis markers in LDL receptor knockout mice, another model of atherosclerosis.^227^ These findings underscore the role of heavy metals in vascular inflammation, oxidative stress, and endothelial dysfunction (*Figure 7*).^253^

#### 6.1.2 Endocrine disruptors

Endocrine-disrupting chemicals, including PFAS, bisphenol A (BPA), and persistent organic pollutants, elevate cardiovascular risks through metabolic dysregulation, oxidative stress, and inflammation^254,255^ (*Figure 7*). BPA exposure is associated with increased CVD prevalence, hypertension, and HF.^256–258^ Similarly, PFAS compounds contribute to dyslipidemia and atherosclerosis.^259^ Organophosphate pesticides have also been linked to severe cardiac complications, including arrhythmias and cardiac ECG Q-T prolongation.^260^ Furthermore, dioxins, pesticides, and plastic-associated compounds may promote atherosclerosis via common pathophysiological mechanisms.^261,262^ Preclinical studies confirm these associations, with exposure to dioxins, pesticides, and BPA exacerbating atherosclerotic plaque development in ApoE^−/−^ mice.^263–265^

### 6.2 Micro- and nano-plastics

**Figure 8:**
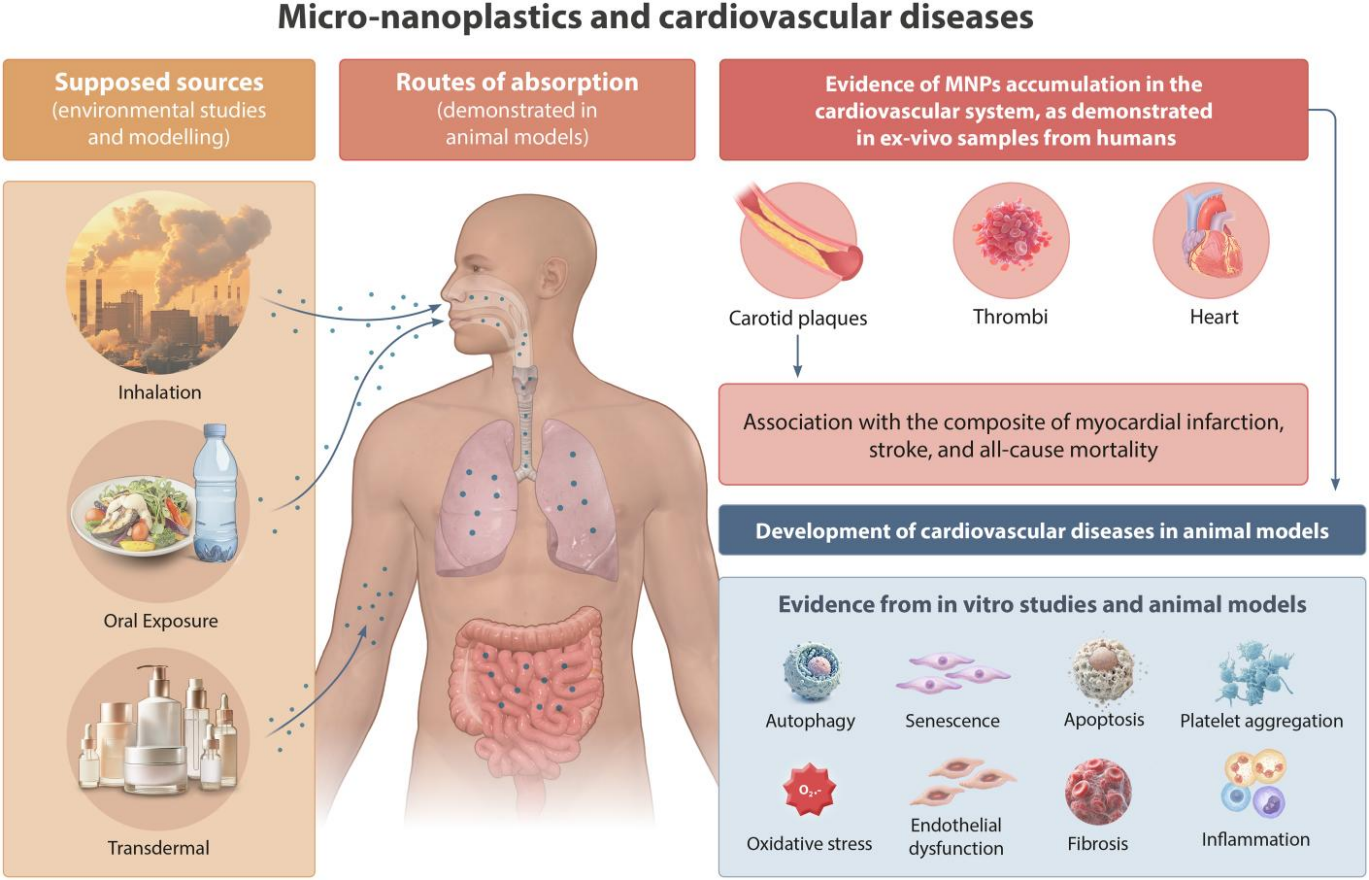
Environmental and modelling studies indicate that micro- and nano-plastics (MNPs) are present in air (indoor and outdoor), water (bottled and tap), food, and cosmetics, making human exposure widespread. As shown in animal models, MNPs can enter the body through inhalation, ingestion, and possibly skin contact. Once in the bloodstream, MNPs may accumulate in cardiovascular tissues, triggering harmful processes such as inflammation, oxidative stress, endothelial dysfunction, and cell damage. Studies on human ex-vivo samples confirm their presence in arterial plaques, with clinical evidence linking MNPs in carotid plaques to increased risks of MI, stroke, and all-cause mortality. (Taken modified from^285^ with permission).

Global plastic production has surged from 2 million tons in 1950 to over 460 million tons in 2019, with waste projected to triple by 2060.^266^ The degradation of plastics generates micro-plastics (≤5 mm) and nano-plastics (≤1000 nm), contaminating soil, water, and marine ecosystems.^267^ Humans are exposed to micro- and nano-plastics (MNPs) primarily through seafood consumption, inhalation, and ingestion of contaminated water.^268,269^ MNPs act as carriers for toxic chemical additives, including phthalates, BPA, PFAS, and heavy metals, further amplifying cardiovascular risk.^270^ MNP exposure induces oxidative stress, inflammation, and vascular dysfunction. Studies show that MNPs trigger endothelial cell senescence by upregulating p53, p21, and p16, contributing to endothelial dysfunction and atherosclerosis.^271^ In preclinical models, MNP ingestion promotes fat accumulation, oxidative stress, and cardiometabolic disease.^272^ Wistar rats exhibit cardiac fibrosis and pyroptosis via the NLRP3/caspase-1 pathway upon MNP exposure.^273,274^ Additionally, MNPs impair nitric oxide signalling and activate inflammatory pathways, exacerbating vascular injury.^275^ A recent study found MNPs in carotid atheromas in humans, the presence of which was linked to a 4.5-fold increased risk of MI, stroke, and cardiovascular mortality.^276^ Higher plastic particle numbers in plaques also correlated with elevated inflammatory markers, including interleukin-6, TNF-α, and CD68. Emerging evidence suggests MNP deposits in various vascular beds, further supporting their role in atherosclerosis.^277^ Moreover, MNPs promote prothrombotic effects and haemolysis, increasing cardiovascular complications.^278,279^ Preclinical data support these human findings, with polystyrene nano-plastics exacerbating atherosclerosis in ApoE−/− mice.^280,281^ MNPs also stimulate vascular smooth muscle cell proliferation, accelerating atherosclerotic lesion formation.^282,283^ Rigorous research is required to further ascertain the risks posed by MNPs, especially in terms of specific methods to detect MNPs in biological specimens and the use of environmentally relevant MNPs in toxicological studies.^284^ Nonetheless, these studies highlight MNPs as emerging cardiovascular risk factors with significant implications for public health (*Figure 8*).

## 7. Mitigation measures

### 7.1 Air pollution

Air pollution, predominantly from fossil fuel combustion, is a leading environmental risk factor for CVD, responsible for an estimated 8.3 million premature deaths annually worldwide, according to Lelieveld *et al*.^9^ Of these, 5.1 million deaths could be prevented by phasing out fossil fuels, underlining the immense potential for cardiovascular health gains through energy system transformation.^286^

To mitigate the cardiovascular burden of air pollution, comprehensive action is needed across both societal and individual levels. At the global scale, transitioning to a 100% renewable energy system—dominated by solar and wind—would lead to an 83–99% reduction in major pollutants (NO_x_, SO_x_, PM_2.5_, PM_10_) by 2050.^287^ Such a transition would also reduce greenhouse gas emissions and slow climate change, thus producing a double benefit.

Adherence to air quality guidelines is central to pollution mitigation. The WHO's 2021 guidelines suggest to limit annual mean PM_2.5_ to <5 µg/m^3^ and NO_2_ to 10 µg/m^3^—levels linked with minimal CVD risk.^213^ However, most urban areas exceed these guidelines, underscoring the need for stricter national air quality standards and enforcement, especially in highly polluted regions like South Asia, where cardiovascular mortality from pollution is disproportionately high.

In addition to societal reforms, personal-level strategies can offer significant protection, particularly in high-exposure settings. Rajagopalan *et al*.^288^ recommend evidence-based measures such as:

- Using high-efficiency particulate air filters indoors, which can reduce indoor PM_2.5_ levels by up to 60%.
- Wearing N95 masks during episodes of high outdoor pollution or while commuting, which would reduce PM exposure.
- Avoiding or limiting outdoor activity during peak pollution hours, particularly near traffic-heavy areas.
- Dietary interventions rich in antioxidants and omega-3 fatty acids may mitigate pollutant-induced oxidative stress and inflammation.
- Optimising cardiovascular risk management, including tight control of hypertension, diabetes and dyslipidemia, to enhance resilience to air pollution's effects.

These personal actions, while beneficial, are not substitutes for regulatory or structural mitigation. IT is important to re-emphasize that mitigating the cardiovascular effects of air pollution requires a dual approach: urgent structural reforms to phase out fossil fuels and meet WHO air quality limits, alongside personal strategies to reduce exposure, especially in those who may be particularly vulnerable and/or susceptible. Together, these measures can deliver profound health benefits, save millions of lives, and reduce the global economic burden of pollution, aligning with both public health and climate goals.

### 7.2 Noise exposure

Local authorities can mitigate noise from roads, railways, and aircraft through various strategies (for review see^111^). For road traffic, noise primarily comes from the tire-road contact at speeds above 30–35 km/h for cars and 55–65 km/h for heavy vehicles. Therefore, transitioning to an electric car fleet will only result in minor noise reductions, if any. As electric cars often weigh more than cars with a combustion engine, they are likely to emit higher levels of rolling noise. Effective measures include noise barriers (up to 10 dB(A) reduction), noise-reducing asphalt (3–6 dB(A)), and speed limit reductions (∼1 dB(A) per 10 km/h decrease). Developing low-noise tires could lower noise by 2–3 dB(A) nationwide, although care will be needed to ensure that material used does not cause airborne tire wear PM to have more toxicity. Urban infrastructure investments—promoting biking, ride-sharing, and public transport—can also reduce noise. Since individual measures yield modest reductions, combining strategies is essential in densely populated areas. For aircraft, optimising air traffic routes via GPS guidance reduces noise over urban areas. Also, night flight bans significantly cut nighttime aircraft noise—a time-window known to be particularly harmful to CVD health. A continuous descent approach, with steeper and smoother landings, a continuous descending approach minimizes noise impact. For railway noise, key strategies include rail grinding to reduce track wear and noise, upgrading brakes to quieter composite materials, and restricting nighttime operations near residential zones. These combined efforts can significantly reduce transportation-related noise pollution.

### 7.3 Heat and wildfires

#### 7.3.1 Mitigating the health risks of extreme heat

Public health interventions are essential to reduce heat-related cardiovascular mortality. Cooling strategies, improved air conditioning, and public awareness campaigns should be expanded, such as those of the U.S. CDC's Climate and Health Program.^289^ Home monitoring of weight, blood pressure, and symptoms of heat-related illness can help prevent complications. Urban planning should address heat islands by increasing green spaces, using reflective roofing materials, and enhancing city tree coverage, which could prevent thousands of premature deaths.^290,291^ Heatwaves disproportionately affect low-income and marginalized populations, particularly in regions least responsible for greenhouse gas emissions.^168^ Effective public health policies and interventions are essential to mitigate these risks and protect vulnerable populations. In addition, as climate change intensifies extreme temperatures, incorporating cardiovascular health considerations in particular concerning city design will become increasingly necessary.

#### 7.3.2 Protecting cardiovascular health from wildfires

Mitigation strategies are crucial for reducing wildfire-related cardiovascular risks. Forecasting high-risk events, educating patients, and adjusting medication regimens during smoke episodes can minimize health impacts.^212^ Indoor air filtration, designated clean air shelters, and adequately fitted N95 masks effectively protect against smoke inhalation.^292^ Given the increasing prevalence of wildfires, further research is needed to understand their long-term cardiovascular effects fully.

### 7.4 Heart-healthy city design

Urban areas remain hotspots for environmental stressors, including climate change, air pollution, noise, light pollution, and heat from urban heat islands.^293^ Additional risk factors such as crime, limited green spaces, social isolation, prolonged sitting, sedentary lifestyles, and poor nutrition contribute to the burden of NCDs. Physical inactivity alone accounts for 70 million DALYs and 3.2 million deaths annually.^293^

Compact cities, characterized by high density, shorter travel distances, and diverse land use, are promoted for sustainability and public health benefits. Increased active transportation, such as walking and cycling, reduces CO_2_ emissions and enhances fitness, and reduces CVD risk.^293^ However, a study of 1000 European cities found that very densely populated compact cities also experience higher air pollution, intensified heat island effects, reduced green spaces, and elevated mortality rates.^291^ Boston, USA, and Melbourne, Australia, with 80 and 85% car-dependent transport, respectively, could significantly benefit from alternative land use and transport policies.^293^ While compact cities offer benefits, poor planning can lead to adverse health outcomes. For instance, Barcelona, Spain, despite its compact structure, still faces high air pollution and traffic-related risks. Currently, Barcelona allocates 60% of public space to cars, despite only 25% of transportation involving motor vehicles.^294^ Policies to reduce traffic density, enhance air quality, and expand green spaces can lower mortality rates and disease burdens.^295^

Innovative urban planning concepts, including Superblocks, low-traffic neighbourhoods, 15-min cities, and car-free models, aim to reduce car dependency and enhance green infrastructure. These strategies improve air quality, lower noise pollution, mitigate heat island effects, and promote physical activity, benefiting cardiovascular health.^294^ Reducing car dominance allows for more parks, cycling paths, and pedestrian-friendly spaces. The 15-min city model, implemented in Paris, prioritizes access to work, education, shops, entertainment, and social activities within a short walk or bike ride, fostering a healthier lifestyle.^296^ Barcelona’s plan to create 500 Superblocks limits motorized traffic within designated areas, promoting green spaces, social interactions, and economic activity. These efforts aim to prevent up to 700 premature deaths annually by improving air quality, reducing noise pollution, preventing heat islands, and increasing physical activity.^291^ Similarly, low-traffic neighbourhoods can be implemented quickly through streetscape changes, making cities safer for walking and cycling while reducing traffic-related injuries and air pollution. Hamburg aims to become a car-free city by 2034, responding to climate change and public health needs. Car-free neighbourhoods, such as Vauban in Freiburg and Pontevedra in Spain, demonstrate the viability of pedestrian-friendly urban models with low CO_2_ emissions. Utrecht's Merwede district in the Netherlands, designed for 12 000 residents, follows a similar approach.^297^ These models alleviate air pollution-related health burdens, promote active transportation, and enhance urban liveability.

Long-term urban planning efforts require complementary short-term policies. Measures such as 30 km/h speed limits and ultra-low emission zones significantly improve public health by reducing accidents and air pollution.^298,299^ Tactical urbanism—temporary, cost-effective urban improvements—can also rapidly transform public spaces and pilot new infrastructure designs. Fossil fuel reliance for energy and transportation remains a primary source of air pollution and climate change.^300^ In 2019, only 0.18% of the global land area had PM_2.5_ exposure below WHO's 5 μg/m^3^ guidelines,^301^ The largest urban PM_2.5_ contributors include energy production, transportation, industry, and residential heating.^212^ Electrification of transport and renewable energy adoption can reduce both greenhouse gas emissions and air pollution, yielding significant health benefits.^302^ However, reliance on biofuels and biomass burning could pose additional risks, as some fuels appear to generate more toxic PM_2.5_ than fossil fuels.^212^ Green spaces mitigate urban environmental risks by reducing air pollution, noise, and heat while promoting physical activity and mental well-being.^303,304^ Studies suggest that green areas’ proximity, size, and connectivity influence cardiovascular health benefits.^305^ Moreover, equitable access to green spaces is essential, as low-income neighbourhoods often lack high-quality parks.^306^

Urban heat islands, caused by heat-absorbing surfaces and reduced vegetation, increase cardiovascular-related mortality.^307^ A study across 93 cities found that urban heat islands raise temperatures by an average of 1.5°C, causing 6700 premature deaths annually. Increasing tree coverage to 30% could lower city temperatures by 0.4°C and prevent 2644 deaths.^291^ Transportation infrastructure impacts CVD through air pollution, noise, stress, and inactivity. Globally, 1 in 4 adults and 3 in 4 adolescents fail to meet WHO physical activity recommendations.^308^ Active transportation—walking and cycling—improves cardiovascular health, yet urban planning must ensure safe infrastructure to maximize benefits while mitigating pollution exposure.^309^

Urban food environments influence cardiovascular risk beyond diet quality. Food insecurity, stress from economic hardship, and exposure to air pollution from food transport contribute to health disparities.^310,311^ Policies promoting healthier food systems include reducing sugar-sweetened beverage sales, supporting local farmers’ markets, and integrating sustainability goals into urban planning.^312^ Contaminated urban water supplies and inadequate waste management expose populations to harmful metals and chemicals linked to CVD.^313^ Substances such as lead, cadmium, and per- and PFAS disrupt cardiovascular function.^314^ Solid waste mismanagement further exacerbates pollution and environmental degradation, disproportionately affecting marginalized communities.^315^ Implementing sustainable water and waste policies is crucial for improving urban public health.^316^

Thus, heart-healthy urban planning must integrate sustainable mobility, green spaces, energy-efficient systems, and equitable infrastructure. Compact cities, low-traffic neighbourhoods, and car-free models offer promising solutions but require careful implementation to minimize unintended health risks. Policy interventions, including speed limits, emission zones, and urban greenery expansion, can provide immediate health benefits. Addressing climate change, pollution, food security, and waste management is integral to fostering resilient and healthy cities.

## 8. Calculating cardiovascular risk by using the exposome

**Figure 9:**
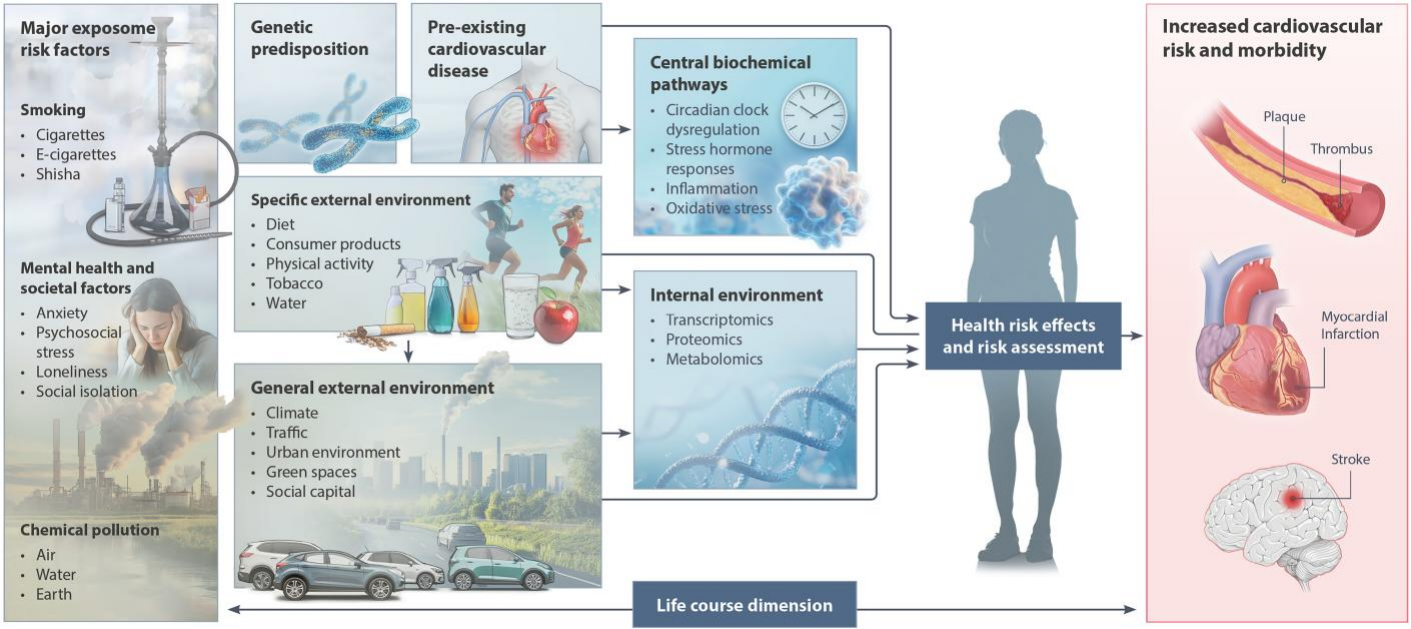
The exposome encompasses all lifelong environmental exposures, their impact on biochemical pathways, and related health effects. The most significant environmental health risk in the general external environment is chemical pollution, while in the specific external environment, tobacco smoking and unhealthy diets have the greatest impact. Mental health can also be affected by environmental factors like social isolation and work strain, though their contribution to disease burden may be underestimated due to limited exposure–response data. Exposures influence the internal environment, often measured through transcriptome, epigenome, proteome, and metabolome changes. Key biochemical alterations include circadian dysregulation, stress hormone release (cortisol, catecholamines), oxidative stress from mitochondria and immune cells, inflammation, and oxidative tissue damage. Environmental exposures can act synergistically with genetic predisposition or existing CVD, worsening outcomes such as atherosclerosis, vascular stenosis, MI, heart failure, and stroke. Taken modified from^114^ with permission.

CVD remains the leading cause of mortality worldwide, with risk factors traditionally classified into modifiable (e.g. smoking, hypertension, diabetes, dyslipidemia) and non-modifiable (e.g. age, sex, genetic predisposition) categories. While these traditional risk factors provide a strong foundation for estimating cardiovascular risk, and their application has saved many millions of lives, they fail to account for the complex interactions between environmental exposures and biological responses over an individual's lifetime. The concept of the exposome, which encompasses the totality of environmental exposures from conception onward, is essential for accurately quantifying cardiovascular risk in a modern and holistic manner (*Figure 9*).

### 8.1 Limitations of traditional cardiovascular risk factors

Traditional cardiovascular risk prediction models such as the Framingham Risk Score or SCORE have significantly contributed to CVD prevention. However, these models predominantly focus on lifestyle and genetic predispositions while neglecting environmental factors, which have become increasingly significant contributors to cardiovascular health. The GBD highlights that factors such as air pollution, noise, chemical exposure, and socioeconomic stressors play a critical role in the pathogenesis of CVD, yet they remain under-represented in conventional risk assessment frameworks.^172,317^

### 8.2 The exposome: a comprehensive approach to cardiovascular risk

Christopher Wild introduced the exposome concept in 2005.^318^ It integrates all environmental factors an individual encounters throughout life, including chemical pollutants, social determinants, lifestyle choices, and psychosocial stressors. By examining these exposures about genetic susceptibility and biological responses, the exposome provides a more precise and individualized assessment of cardiovascular risk (*Figure 9*).

1. Multi-Exposure Synergy: Traditional models assume isolated risk factors, yet environmental exposures rarely occur in isolation. Urban environments expose individuals to a combination of air pollution, noise, heat, and social stressors, which together exert a cumulative and potentially synergistic impact on cardiovascular health.^319^
2. Biological Pathways and Mechanisms: Exposome research enables the identification of novel biomarkers and molecular pathways linking environmental exposures to CVD. For example, air pollution triggers oxidative stress pathways that accelerate atherosclerosis, while chronic noise exposure disrupts circadian rhythms and elevates stress hormones, contributing to hypertension.^320^
3. Precision Medicine and Public Health Interventions: The exposome allows for personalized risk assessments by integrating genetic susceptibility with real-world exposures. This can guide targeted interventions such as urban planning to reduce pollution, noise regulations, and policies aimed at minimising occupational and socioeconomic stressors.^321^

Thus, to achieve a truly precise cardiovascular risk estimation, we must move beyond traditional risk factors and adopt an exposomic approach. Incorporating environmental determinants into cardiovascular risk prediction models will enhance individual risk assessment and inform public health strategies to mitigate the growing burden of CVD in the modern world.^15^

## 9. The detrimental costs of inaction:

The economic consequences of failing to address environmental exposures—particularly air pollution and noise—are staggering and extend far beyond healthcare. In the United States alone, calculations show that fossil fuel-related air pollution contributes to at least 107 000 premature deaths annually, incurring health-related costs exceeding $820 billion per year.^322^ Globally, the World Bank estimated the cost of inaction on air pollution in 2019 to be $8.1 trillion, representing 6.1% of global GDP guide to assessing. Within the European Union, research suggests that the social cost of noise and air pollution in the EU—including death and disease—could be nearly €1 trillion. For comparison, the social cost of alcohol in the EU has been estimated to be €50–120 billion and smoking at €544 billion—however, investments in clean air yield significant economic returns.^323^ For instance, the U.S. Clean Air Act was estimated to have a benefit-cost ratio of approximately 30:1, with the vast majority of benefits arising from reduced mortality due to improved air quality.^324^ In the UK, introducing clean air zones, such as in Bradford, has been estimated to already have led to monthly National Health Service savings exceeding £30 000 and reductions in respiratory and cardiovascular morbidity.^325^ Similarly, London's Ultra Low Emission Zone in the UK is projected to prevent over 1.4 million air pollution-related hospital admissions by 2050.^326^ Beyond health, improved air quality also enhances urban economic value—cities that achieve a 10% improvement in air quality report a 5.6% increase in property values, with projected capital gains of over $60 billion in the U.S. alone.^327^ Moreover, clean air action is cost-effective in terms of productivity: workplace exposures to PM_2.5_ and heat are associated with substantial losses in labour output, particularly in lower-income and industrial sectors.^328^ Despite these straightforward returns on investment, clean air initiatives still receive only 1% of international development funding,^329^ a discrepancy that underscores the need to prioritize environmental health in national and global policy agendas. Recent modelling studies by Lelieveld *et al*.^9^ show that a 50% phase out of fossil fuel combustion could eliminate up to 82% of premature deaths from air pollution, demonstrating the economic and health imperative of swift and systemic mitigation efforts.^9^

In addition, inaction on noise and soil and water pollution impose significant economic burdens through rising rates of CVD and associated healthcare costs. In the United States, the total healthcare costs attributable to man-made pollution—including contaminated air, water, and soil—range between $240 billion and $883 billion annually, with a substantial portion related to cardiovascular outcomes.^322^ In Europe, exposure to PFAS, known as ‘forever chemicals,’ incurs estimated health costs of €52–84 billion annually.^330^ Also, recent findings estimate that removing toxic chemicals from plastics would yield substantial health and economic benefits by reducing the burden of CVD and other diseases.^258^ In addition, lead exposure is expected to be responsible for over 5.5 million cardiovascular deaths annually and $6 trillion in economic losses, equivalent to 7% of global GDP.^10^ Lastly, the EEA has found transport noise to result in 40 000 new cases yearly of IHD in EU annually, a number that is expected to be underestimated for CVDs; including other CVDs (stroke, HF, and CVD mortality), a lower threshold than the current calculation threshold of 55 dB, and nationwide noise calculations will increase these numbers significantly.^331,332^

When compared to other environmental health threats, transport noise ranks among the top three—just behind air pollution and temperature-related factors. Chronic exposure to noise from transport contributes to 66,000 premature deaths annually in Europe, while also leading to around 50,000 new cardiovascular disease cases and 22,000 cases of type 2 diabetes.^333^ These findings emphasize the urgent need for preventative action to mitigate the cardiovascular and economic consequences of air, noise, soil, and water pollution.

## 10. Gaps in knowledge

Despite significant advancements in understanding the impact of environmental risk factors on CVD, substantial gaps remain in mechanistic insights and epidemiological evidence. Addressing these gaps is crucial for developing targeted interventions and public health strategies. The long-term effects of noise pollution are not fully understood, particularly the cumulative effects of lifelong exposure. While acute noise exposure is known to impair vascular function and increase stress hormone levels, the potential for irreversible cardiovascular damage due to chronic exposure remains unclear. Additionally, the interplay between noise and other environmental stressors such as air pollution, artificial light at night, and climate factors is not well studied, limiting our understanding of their combined effects. Individual susceptibility, influenced by genetic predisposition, pre-existing conditions, and socioeconomic factors, also requires further research to identify vulnerable and susceptible populations.

The cardiovascular effects of air pollution at low doses remain uncertain. While high levels of PM_2.5_ and UFPs are established risk factors, the impact of exposure below current regulatory limits needs further investigation. Air pollution rarely occurs in isolation, yet studies on co-exposure to different pollutants, such as PM_2.5_ with nitrogen oxides, remain scarce. The dominant hypothesis attributes air pollution-induced CVD to oxidative stress and inflammation, but other pathways, including epigenetic modifications and microbiome alterations, need exploration. Emerging pollutants, such as microplastics and novel industrial chemicals, require urgent attention due to their increasing presence in air, water, and food, with unknown cardiovascular consequences. We also want to have a better assessment of the CVD benefits of interventions reducing air pollution levels.

Climate change and extreme weather events pose additional risks. The physiological mechanisms linking heat waves to cardiovascular mortality are not fully understood, particularly concerning the modulatory effect of medications and hydration status. The cardiovascular effects of desert dust storms and wildfire smoke, which have unique chemical properties, are underexplored, especially in terms of the toxicity of air-assimilated constituents from sources that are anthropogenic in origin. Artificial light at night and circadian disruption have been linked to hypertension and metabolic syndrome, but the biological pathways remain unclear. Research on chemical pollutants, including endocrine disruptors and heavy metals, is limited, particularly regarding chronic exposure at low levels.

Urban planning interventions, such as compact cities and green spaces, hold promise but require evaluation for direct cardiovascular benefits. Many mitigation strategies lack long-term assessments. Addressing these knowledge gaps through interdisciplinary research is essential to mitigate the cardiovascular burden of environmental risk factors.

Significant uncertainties surround the contribution of manufactured chemicals to CVD incidence and mortality. A major shortcoming that limits the assessment of the disease burden due to manufactured chemicals is that most of the many thousands of manufactured chemicals in commerce have never been tested for toxicity. Without even the most basic information on the potential toxicity of these widely used materials, it is impossible to estimate the magnitude of their harms to health. A fundamental revision of chemical safety legislation to require toxicity testing of all chemicals in commerce will be required to rectify this situation and improve health. It has been proposed that chemicals that result in human exposure should be subjected to the same degree of regulatory scrutiny as pharmaceutical chemicals.^334^

Despite growing concern over micro- and nanoplastics (MNPs), there is a striking absence of epidemiological evidence linking them to CVD. While preclinical studies show that MNPs can trigger oxidative stress, inflammation, and endothelial dysfunction—mechanisms central to CVD—no population-based studies have yet assessed these effects in humans. This lack of data is concerning given the widespread presence of MNPs in air, water, food, and even human blood. The epidemiological silence on MNPs represents a critical knowledge gap and highlights the urgent need for well-designed studies to evaluate their potential role in cardiovascular morbidity and mortality.

## 11. Major conclusions and resulting political/societal needs for action

This comprehensive review of environmental risk factors underscores the reality that environmental risk factors are major but insufficiently appreciated risk factors for CVD. The findings we present here make it clear that selected environmental exposures need to be added to the list of clinical and behavioural risk factors that physicians routinely consider in evaluating CVD risk in their patients. The evidence underscores the urgent need for targeted public health interventions and policy actions. Individual interventions and behavioural change are not sufficient to address these risks. These factors contribute to a substantial global disease burden, necessitating immediate and coordinated action at a societal level across disciplines and policy sectors.

A key conclusion from this review is the necessity of stringent regulatory measures to mitigate exposure to these environmental hazards. Stricter air quality standards should be implemented to limit PM_2.5_ and nitrogen oxides, by formulating roadmaps to implement the WHO guidelines. Innovative methods are needed to measure UFPs and volatile organic compounds at scale, particularly in urban areas where population exposure is highest, and design guideline levels in line with improved data on their toxicity. The adoption of ultra-low emission zones, expansion of public transportation, and electrification of vehicle fleets can significantly reduce pollution-related cardiovascular risks. While electric vehicles will still produce PM from brakes, tires and road wear, and more research is needed to determine the exposure and toxicity of these PM, eliminating the known harm of vehicle tailpipe emissions would assuredly improve health. Noise pollution remains an underappreciated yet critical contributor to CVD. Stronger policies are required to regulate transportation noise, including nighttime aircraft restrictions, improved urban planning to minimize residential exposure, and the implementation of noise barriers. Additionally, environmental noise, and other environmental risk factors, should be integrated into cardiovascular risk assessments to better inform medical and public health recommendations. The increasing recognition of artificial light at night as a cardiovascular risk factor necessitates policy changes to reduce light pollution. This includes curfews on excessive outdoor lighting, modifying streetlights to minimize blue light emissions, and restricting advertising billboards that contribute to light exposure in urban settings.

Climate change is an escalating public health crisis that exacerbates environmental risks for CVD. Policies to combat climate-related health threats should prioritize urban cooling strategies, such as increasing green spaces, implementing reflective roofing, and strengthening early warning systems for extreme heat events. Wildfire smoke, desert dust storms, and extreme temperature events must be integrated into national health policies with adaptive strategies to protect vulnerable and susceptible populations, including individuals with preexisting CVD.

Public health campaigns should increase awareness of the cardiovascular risks of environmental stressors, advocating for lifestyle modifications such as physical activity, improved diet, and stress management to mitigate exposure effects. Additionally, interdisciplinary research should be expanded to close existing knowledge gaps, particularly concerning the long-term impact of combined exposures and individual susceptibility. To reduce the cardiovascular burden of environmental risk factors, governments must adopt proactive and enforceable policies that prioritize public health, environmental sustainability, and equitable access to protective measures. Integrating environmental determinants into CVD prevention strategies is essential to reducing morbidity and mortality on a global scale.
